# Disentangling clustering configuration intricacies for divergently selected chicken breeds

**DOI:** 10.1038/s41598-023-28651-8

**Published:** 2023-02-27

**Authors:** Anatoly B. Vakhrameev, Valeriy G. Narushin, Tatyana A. Larkina, Olga Y. Barkova, Grigoriy K. Peglivanyan, Artem P. Dysin, Natalia V. Dementieva, Alexandra V. Makarova, Yuri S. Shcherbakov, Marina V. Pozovnikova, Yuri V. Bondarenko, Darren K. Griffin, Michael N. Romanov

**Affiliations:** 1 Russian Research Institute of Farm Animal Genetics and Breeding – Branch of the L. K. Ernst Research Science Center for Animal Husbandry, Pushkin, St. Petersburg, Russia; 2 Research Institute for Environment Treatment, Zaporozhye, Ukraine; 3 Vita-Market Ltd, Zaporozhye, Ukraine; 4 Sumy National Agrarian University, Sumy, Ukraine; 5 School of Biosciences, University of Kent, Canterbury, UK; 6 L. K. Ernst Federal Research Centre for Animal Husbandry, Dubrovitsy, Podolsk, Moscow Oblast Russia

**Keywords:** Agricultural genetics, Animal breeding, Genetic markers, Genome, Genetic variation, Structural variation, Genomics, Comparative genomics, Genotype, Genetics, Population genetics, Genetic variation, Applied mathematics, Computational models, Data integration, Functional clustering, Phylogeny

## Abstract

Divergently selected chicken breeds are of great interest not only from an economic point of view, but also in terms of sustaining diversity of the global poultry gene pool. In this regard, it is essential to evaluate the classification (clustering) of varied chicken breeds using methods and models based on phenotypic and genotypic breed differences. It is also important to implement new mathematical indicators and approaches. Accordingly, we set the objectives to test and improve clustering algorithms and models to discriminate between various chicken breeds. A representative portion of the global chicken gene pool including 39 different breeds was examined in terms of an integral performance index, i.e., specific egg mass yield relative to body weight of females. The generated dataset was evaluated within the traditional, phenotypic and genotypic classification/clustering models using the *k*-means method, inflection points clustering, and admixture analysis. The latter embraced SNP genotype datasets including a specific one focused on the performance-associated *NCAPG*-*LCORL* locus. The *k*-means and inflection points analyses showed certain discrepancies between the tested models/submodels and flaws in the produced cluster configurations. On the other hand, 11 core breeds were identified that were shared between the examined models and demonstrated more adequate clustering and admixture patterns. These findings will lay the foundation for future research to improve methods for clustering as well as genome- and phenome-wide association/mediation analyses.

## Introduction

The global chicken gene pool has been shaped during thousands of years of domestication and demographic history of diverse chicken breeds. These meet versatile human needs for table eggs, poultry meat and aesthetic preferences, culminating in a wide variety of chicken breeds with valuable genomic and phenomic features. They have arisen on different continents and in different countries as a consequence of artificial selection for certain phenotypic (productive) traits and specialized interbreeding (e.g.,^1–5^). Moiseyeva et al.^6^ established four major evolutionary lineages of chicken breed formation: (1) egg type (ETB), (2) meat type (MTB), (3) game (GB), and (4) Bantam (BTB; or miniaturized type) breeds. Comparing the phenotypic and genotypic features of a large sample of the world gene pool, Larkina et al.^7^ added two more chicken breed formation categories: dual purpose (DPB), including egg-meat (EMB) and meat-egg (MEB) subtypes, and fancy (FB; or ornamental) breeds.

In our previous studies^7,8^, we considered three main classification (clustering) models for the evolutionarily determined subdivision of the global chicken breed gene pool. These were: (1) traditional classification model (TCM) generally accepted in poultry breed categorization; (2) phenotypic clustering model (PCM) built according to a suite of phenotypic/performance traits; and (3) genotypic clustering model (GCM; including its two variants, GCM1 and GCM2) based on single nucleotide polymorphism (SNP) genotypes at the well-known *NCAPG*-*LCORL* locus associated with chicken performance^7,9–11^. This locus has been identified in mammals as a locus associated with body growth and development. Its significant associations with height were shown for the Liangzhou donkey^12^, cattle^13^, as well as in relation to body weight and skeletal size in sheep^14,15^. Significant SNPs at this locus appear to influence egg weight^16^, oviduct size^17^ and internal organ mass in chickens^18^. With some preference in favor of PCM, it was, however, very difficult to decide unambiguously which of the classification (clustering) models of breeds was the most suitable.

In cluster analysis, especially when looking for plausible distribution configurations of species, breeds or populations, one often turns to the use of *k*-means clustering^19–22^ as well as the elbow method of clustering (e.g.,^23^). A nonhierarchical *k*-means technique is a popular method of multivariate analysis^24^, which was also used in cluster analysis to describe the egg-laying patterns of hens (e.g.,^25^). This algorithm seeks to minimize the total square deviation of cluster points from these clusters’ centers^21^. The elbow method is a heuristic used in determining the number of clusters in a dataset by plotting the explained variation function and picking the optimal number of clusters at the elbow point of the explained variation curve (e.g.,^26,27^). The elbow method is also applicable for inferring ancestral populations in the admixture analysis based on multi-locus SNP genotypes^28^. For instance, Larkina et al.^7^ and Abdelmanova et al.^29^ applied it to choosing the optimal number of clusters (ancestral populations) for interpretation of chicken breed clustering.

In this regard, we set ourselves the goal of testing and improving the well-known clustering algorithms based on, or including, the *k*-means and elbow methods. We also, where possible, established novel algorithms to discriminate between various chicken breeds. Using 39 breeds representing a fairly large portion of the world chicken gene pool, i.e., ~ 6% of the FAO estimate of known chicken breeds^30^, we analyzed the respective datasets for the above three classification (clustering) models described in the previous study^7^. This enabled generation of new insights into clustering configuration intricacies for divergently selected chicken breeds that can be useful in future genome- and phenome-related research.

## Methods

### Chicken breeds

A broad sampling of the global chicken gene pool encompassed a total of 759 hens from the 39 breeds (populations) maintained at the Russian Research Institute of Farm Animal Genetics and Breeding (RRIFAGB) bioresource collection farm (Table 1). The 39 populations were purebred, except a meat-type population of three-way hybrids (White Cornish × (Brahma Light × Sussex Light)) bred inter se.

**Table 1:**
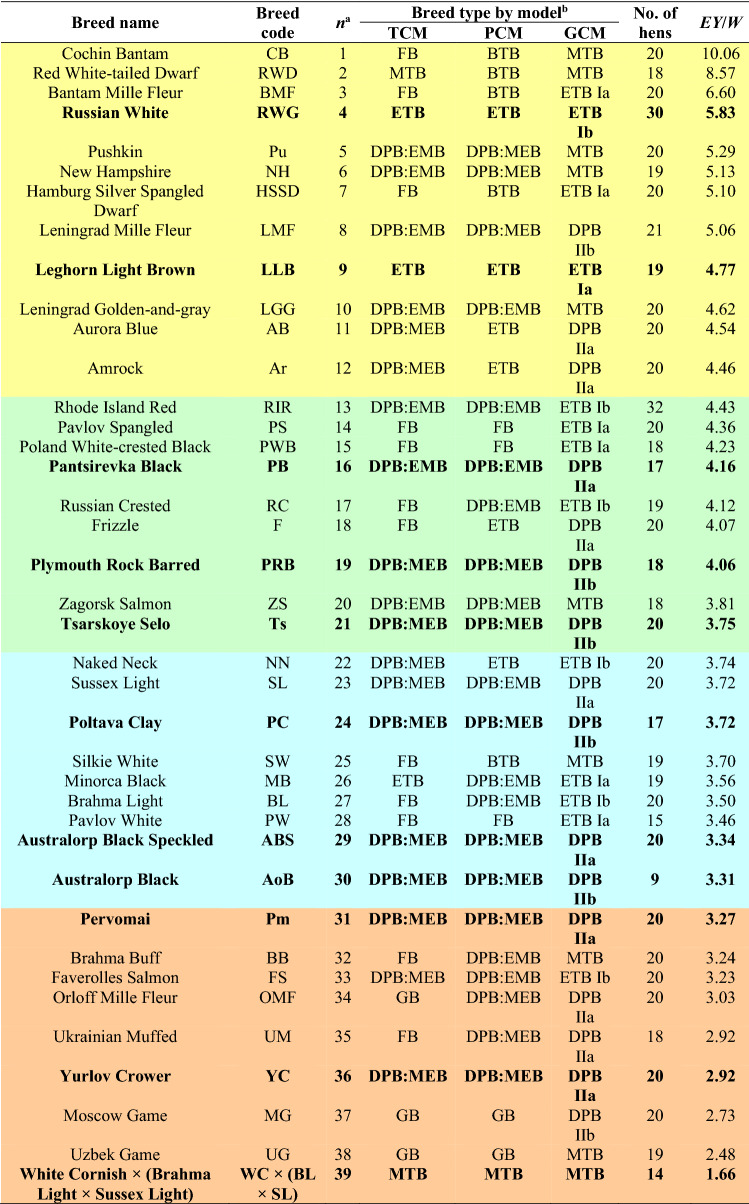
Chicken breeds used in this study respective to breed classification (clustering) models^7^ and ranked by *EY*/*W* values.

^a^*n*-values are conditional serial numbers assigned to chicken breeds according to the degree of descending *EY*/*W* index. ^b^*TCM* traditional classification model, *PCM* phenotypic clustering model, *GCM* genotypic clustering model. Breed types as categorized by Larkina et al.^7^: *ETB* egg, *DPB* dual purpose, *EMB* egg-meat, *MEB* meat-egg, *MTB* meat, *GB* game, *BTB* Bantam, *FB* fancy. Breeds defined as core breeds are shown in bold, and those grouped by the inflection points clustering are highlighted in a different color.

We assessed the following key phenotypic (performance) traits in each breed: mean egg mass yield per hen for 52 weeks of life (*EY*), and mean body weight of sexually mature females at the age of 52 weeks (*W*). *EY*, in turn, was calculated as the product of mean egg weight at 35 weeks of age and egg production for 52 weeks. For subsequent analyses, we introduced an integral performance coefficient for each breed, *EY*/*W*, obtained by dividing *EY* by *W* (Table 1).

For subsequent analysis, we omitted GCM2, since it was the only one of the analyzed models that did not allow us to assess the degree of belonging to ETB among any of the identified performance-based breed types. In GCM1, however, we did not have a breakdown into EMT and MET breeds; therein, we conventionally designated such breeds as DPB^7^. Accordingly, if in the first two models it turned out, for example, that the Pantsirevka Black breed belongs to DPB:EMT breeds, and in GCM1 this was a DPB (Table 1), then we conditionally considered that we had a match of breed types in all three models. For convenience, we further refer to GCM1 as GCM. Venn diagram plotting^31^ was used for visualizing the number of different breed types shared between the three classification (clustering) models, i.e., TCM, PCM, and GCM (Table 1).

### *k*-means clustering

To analyze and show graphically the distribution of chicken breeds for each of the three models, we employed the *k*-means method implemented as a cluster analysis webtool elsewhere^32^. Herewith, values of the aforementioned *EY*/*W* coefficient were used. Within each model, the 39 breeds were grouped and successively tested in three following ways (Supplementary data S1). Firstly, the original arrangement of breeds characteristic of a model was employed, i.e., using their breakdown by a breed type as defined in Larkina et al.^7^. Secondly, the modified model clustering arrangement was applied based on descending sorting by mean *EY*/*W* values per breed type. Thirdly, the modified clustering was used based on descending sorting by the greatest *EY*/*W* values per breed type. That is, a total of three submodel distribution graphs of 39 breeds were plotted for each model as follows: TCM-1, TCM-2, TCM-3; PCM-1, PCM-2, PCM-3; and GCM-1, GCM-2, GCM-3.

To identify in which of the three models/submodels the distribution of breeds by *EY*/*W* values most adequately conformed to the breakdown of breeds into types, the following *k*-means clustering measures were calculated^32^: *k*, number of optimal required clusters identified by the elbow method and tested within this experiment in the range 1 ≤ *k* ≤ 11; SSE (within groups), the sum of square (SS) distances from the points to the cluster centers within breed type groups; SSG (between groups), SS from the cluster centers to the average vector; SST (total), SS from the points to the average vector (i.e., SST = SSE + SSG); mean SSE by group; mean *S* (Silhouette^22^) score; and number of outliers. When considering the *S* score range [–1; 1] for a cluster object, its greatest score (i.e., close to 1) conforms to the situation when the object belongs to its specific cluster. A negative *S* value implies that the object is wrongly assigned to this cluster and misclassified. When a cluster contains only one object, Rousseeuw^22^ suggested that a value of zero may arbitrarily be assigned, i.e., *S* = 0. However, because the cluster analysis webtool^32^ plotted this single object coinciding with its cluster center, we believe that in this case the object completely belongs to its cluster and, therefore, *S* = 1. Consequently, when calculating mean *S* scores for submodels, we used *S* = 1 for single object clusters. This allowed us to overcome a certain bias in submodel *S* scores, if an arbitrary value of zero (as suggested by Rousseeuw^22^) were, otherwise, assigned.

The *k*-means clustering configurations and measures (Supplementary data S1 and S2) were carefully examined to determine for which of the models/submodels the accepted breakdown of breeds into types was most adequately described using the *EY*/*W* coefficient.

### Inflection points clustering

We chose the elbow method of clustering (e.g.,^33^) as a basis of further analysis. Despite the elbow method’s advantages, one can point out at least one essential drawback: such an elbow cannot always be unambiguously detected when employing this heuristic procedure^23,32^.

The elbow method shortcomings can be overcome if (i) a respective clustering index, e.g., an integral coefficient, is chosen and utilized appropriately for producing the breakdown of objects, and (ii) a mathematical algorithm is developed to determine unambiguously the boundaries between clusters. As aforementioned, the ratio of egg production characteristic of layers (i.e., the total mass of eggs produced during a certain laying period) to their weight, *EY*/*W*, served as the integral coefficient. The proposed calculation algorithm resulted in determining the coordinates of the inflection points at which the *EY*/*W*-related function changes the direction of its convexity. To do this, the respective functional dependence had to be approximated by some mathematical function (e.g., a higher-order polynomial), its second derivative being determined and equated to zero (e.g.,^34^). The found values were the desired inflection points of the function, enabling judgement of the boundaries of the corresponding clusters.

### SNP genotyping and admixture analysis

As described in detail elsewhere (e.g.,^7^), genome-wide SNP scanning results generated using an Illumina Chicken 60 K SNP iSelect BeadChip (Illumina, San Diego, CA, USA) were processed with the following PLINK 1.9 program^35^ filters: –geno 0.2, –hwe 0.0001, and –maf 0.05. Out of 57,636 original SNP markers, 44,200 SNPs remained after the filtering. Linkage disequilibrium (LD) between pairs of SNPs was estimated using the *D'* coefficient proposed by Lewontin^36^ and Pearson's *r*^2^ correlation coefficient^37^. Next, we generated admixture models for 11 core breeds using the ADMIXTURE program^28^ (with the preset number of 5 iterations) and SNP genotype data for the whole genome and, separately, for the *NCAPG*-*LCORL* locus. An elbow method-based cross-validation (CV) error plot for determining the number of ancestral populations (K) was produced using Microsoft Excel, with the optimal number being defined using the lowest CV error value from those computed for K = 2 to 6 (using genome-wide SNP dataset) or K = 2 to 6 (at the *NCAPG*-*LCORL* locus). ADMIXTURE bar plots were visualized in RStudio v. 4.1.0^38^ using the pophelper library^39^. To provide additional support to the admixture analysis, principal component analysis (PCA) and phylogenetic analysis were performed. PCA analysis was implemented using PLINK 1.9^35^, and the results were visualized using the ggplor2 library in R^40^. The phylogenetic tree was built on the basis of pairwise genetic distances (*F*_ST_) using the iTOL online service^41^.

### Ethics approval and consent to participate

All experiments complied with the ARRIVE guidelines and were carried out in accordance with the EU Directive 2010/63/EU for animal experiments. The RRIFAGB—Branch of the L. K. Ernst Federal Research Center for Animal Husbandry provided ethical approval for all research using chickens within the framework of the present study (Protocol No. 2020-4 dated 3 March 2020).

## Results

### Overall model assessment

**Figure 1:**
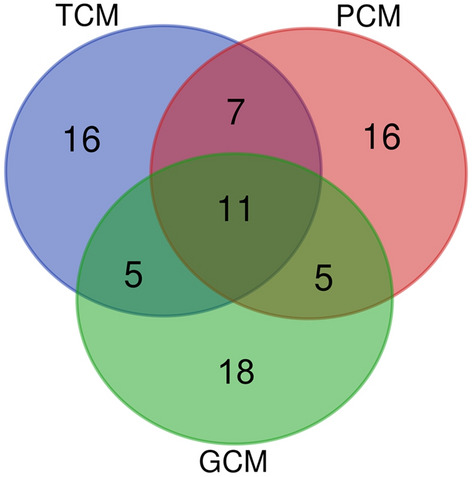
Venn diagram representing distribution of the studied 39 chicken breeds between the three classification (clustering) models: *TCM* traditional classification model, *PCM* phenotypic clustering model, *GCM* genotypic clustering model. Eleven breeds shared between the three models are core breeds.

For the original 39 breeds, three Larkina et al.^7^ models, i.e., TCM, PCM and GCM, were revisited and compared (Table 1). Although there were some discrepancies between the models in classifying (clustering) this breed set, several breeds fall into the same-type or similar classes/clusters in all the three models (as shown in bold in Table 1 and also visualized in the Venn diagram (Fig. 1)).

In other words, particular categories for such breeds were confirmed by all three models, and these were the following 11 chicken populations: Russian White, Leghorn Light Brown (ETB); Pantsirevka Black (EMB); Plymouth Rock Barred, Tsarskoye Selo, Poltava Clay, Australorp Black Speckled, Australorp Black, Pervomai, Yurlov Crower (MEB); and the White Cornish hybrid population (MTB). We conditionally named them *core breeds* (as seen in Fig. 1). For instance, the Russian White breed was included in ETB in each of the three models, the same was true for the Leghorn Light Brown, etc.

### *k*-means clustering

Using the selected appropriate webtool for the *k*-means analysis^32^, we were able to analyze mathematically and express graphically the available model/submodel datasets (Supplementary data S1). A summary of full statistics resulting from the *k*-means clustering is presented in Supplementary data S2. Due to similar breed ranking, GCM-2 and GCM-3 statistics turned out to be identical.

Among the nine submodels (Supplementary data S2), numbers (*k*) of original and optimal required clusters coincided for each of TCM-3, PCM-1 and PCM-3. However, even for them, the plotted cluster configurations differed from the original classification (clustering) models. SSE values ranged between 12.4 (in PCM-3) and 18.4 (in TCM-3). SST values varied from 150.4 (in TCM-2) and 216.7 (in TCM-3). Mean *S* score was the lowest in GCM-1 (0.37 ± 0.18) and the greatest in TCM-3 (0.54 ± 0.22). Pairwise comparison of mean *S* scores resulted in significantly greater *S* values TCM-1 vs GCM-1 (*P* < 0.05), TCM-2 vs GCM-2/GCM-3 (*P* < 0.05), TCM-3 vs GCM-1 and GCM-2/GCM-3 (*P* < 0.001), PCM-1 vs GCM-1 and GCM-2/GCM-3 (*P* < 0.01), PCM-2 vs GCM-1 and GCM-2/GCM-3 (*P* < 0.01), and PCM-3 vs GCM-1 and GCM-2/GCM-3 (*P* < 0.01). Number of cluster outliers per submodel was zero (in PCM-2 and GCM-2/GCM-3) to three (in TCM-1 and GCM-1).

Overall, judging from a total of cluster measures and configurations produced (Supplementary data S1 and S2), GCM submodels seemed to conform to the respective original clustering model to a lesser extent as compared to TCM and PCM submodels. However, none of the submodels looked ideal in this respect.

**Figure 2:**
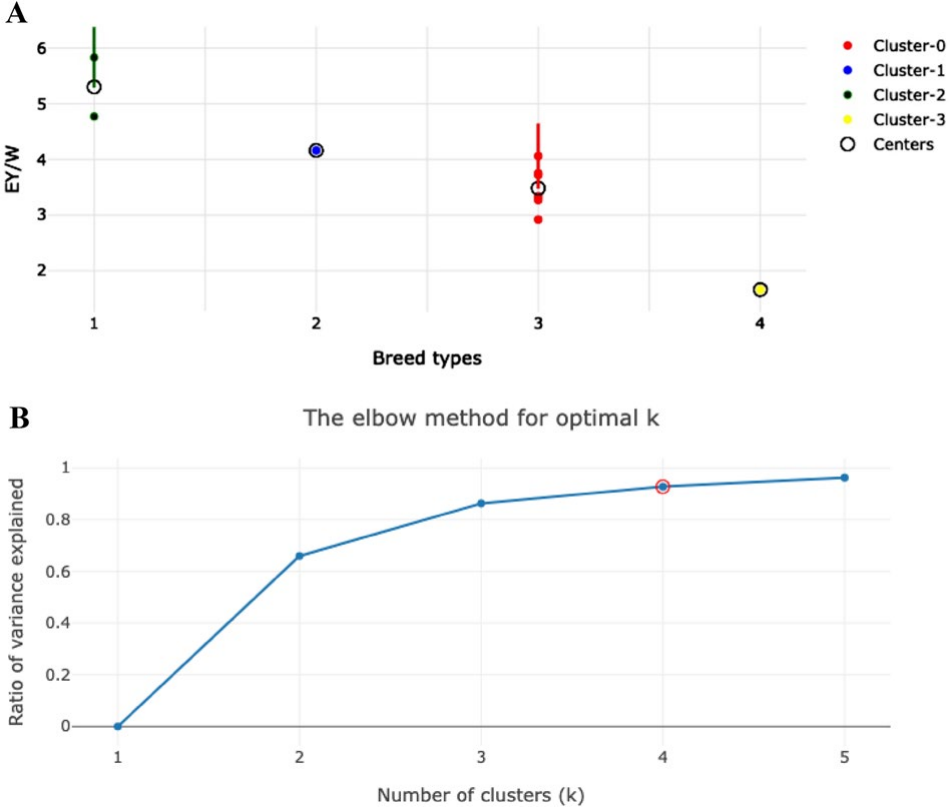
Cluster analysis for distribution of the 11 core breeds using the *k*-means (**A**) and elbow (**B**) methods. Breed (sub)types (**A**): 1, egg type (cluster-2); 2, egg-meat subtype (cluster-1); 3, meat-egg subtype (cluster-0); and 4, meat type (cluster-3). Optimal number of clusters (*k*) was 4 (**B**). *EY*/*W*, integral performance index as a ratio of egg mass yield (*EY*) and female body weight (*W*).

Additionally, we performed the *k*-means clustering analysis for the 11 core breeds produced (Supplementary data S1 and S2). Their distribution almost ideally conformed to the respective breed types (Fig. 2). Mean *S* score for the 11 core breeds (0.6291 ± 0.2677) tended to be greater than those for the 39-breed models TCM and PCM and was significantly greater as compared to GCM (*P* < 0.001; Supplementary data S2). Also, there were no outliers in the 11-breed model.

### Inflection points model

**Figure 3:**
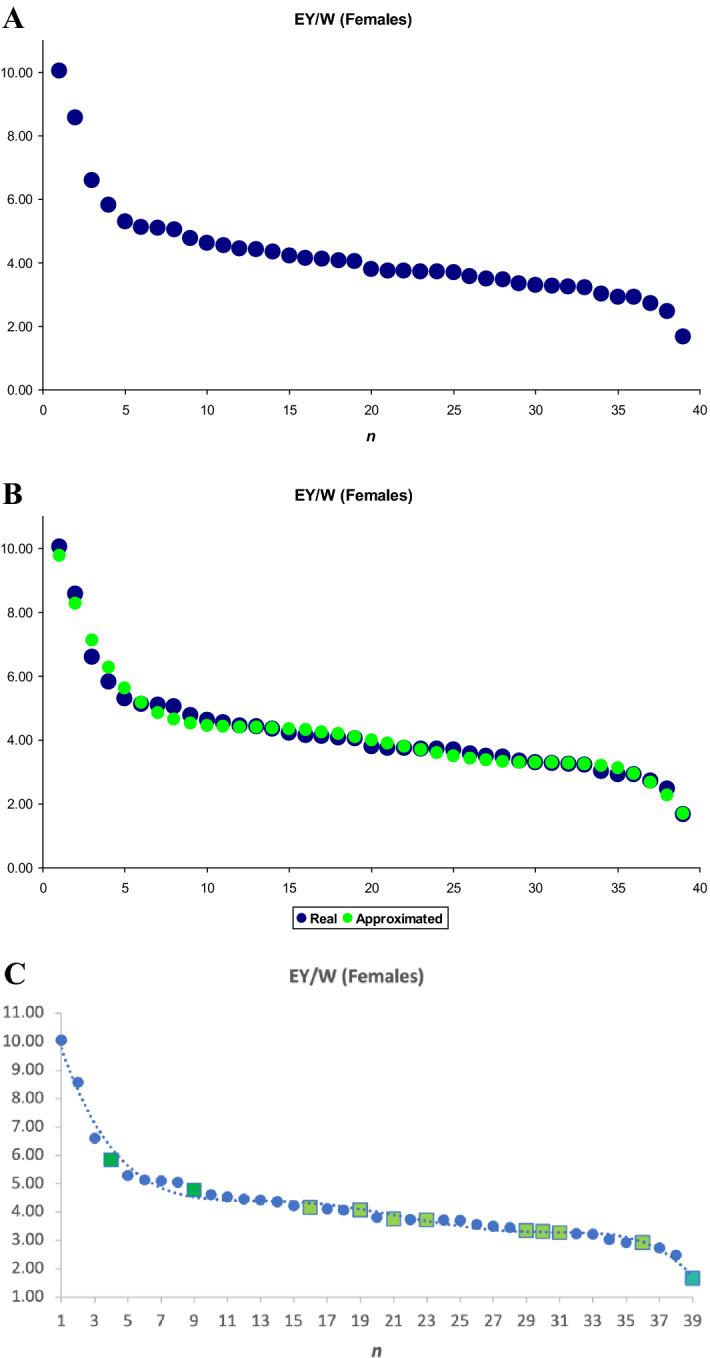
Graph of change in mean *EY*/*W* values in females of the 39 chicken breeds studied. (**A**) original dataset; (**B**) correspondence of the approximated dependence (green trendline) to actual data (blue curve); and **C** original dataset including the respective trendline (blue dotted lines) and 11 core breeds (filled green square). *EY*/*W*, integral performance index as a ratio of egg mass yield (*EY*) and female body weight (*W*); *n* (1 to 39; see Table 1), conditional serial numbers assigned to breeds of chickens according to the degree of descending *EY*/*W* index.

After calculating *EY*/*W*, data for the 39 breeds were ranked from largest to smallest, resulting in chicken breeds arranged in descending order as shown in Table 1 (see also the further details in Supplementary data S3). On the corresponding graph, the pattern of change in *EY*/*W* values looked like that shown in Fig. 3A.

The resulting functional dependence was approximated by the following polynomial:

1 $$\frac{EY}{W} = 11.6480005 - 2.1083905n + 0.23582978n^{2} - 0.0124689n^{3} + 0.00030623n^{4} - 0.00000283n^{5}$$

*R* = 0.992.

The degree of correspondence of the approximate dependence to actual data is shown in Fig. 3b.

Next, we defined the first and second derivatives of Eq. (1):

$$\left( \frac{EY}{W} \right)^{\prime } = - 2.1083905 + 0.47165956n - 0.0374067n^{2} + 0.00122492n^{3} - 0.00001415n^{4}$$

$$\left( \frac{EY}{W} \right)^{\prime \prime } = 0.47165956 - 0.0748134n + 0.00367476n^{2} - 0.0000566n^{3}$$

and equated the second derivative to zero:

$$0.47165956 - 0.0748134n + 0.00367476n^{2} - 0.0000566n^{3} = 0$$

2 $$n^{3} - 64.925088n^{2} + 1321.79152n - 8333.2078 = 0$$

By solving the cubic Eq. (2), the following roots, i.e., inflection points, were found: *n*_1_ = 12.5, *n*_2_ = 21.6, *n*_3_ = 30.8. According to the condition of adequate root definition, the third derivative of Eq. (1) should not be equal to zero. That is,

$$\left( \frac{EY}{W} \right)^{\prime \prime \prime } = - 0.0748134 + 0.00734952n - 0.00016986n^{2} \ne 0$$

Thus, the inflection points were correctly defined.

Collectively, we suggest that the analyzed 39 chicken breeds can be conditionally divided into the following four clusters: 1 to 12, ETB; 13 to 21, EMB; 22 to 30, MEB; and 31 to 39, MTB. In Table 1, each cluster is highlighted with a certain color. Since Larkina et al.^7^ described the three classification (clustering) models that had their own designations (TCM, PCM, and GCM), we can come up with a name and designation for this model, too, suggesting the inflection points model (IPM).

Subsequently, we tried to focus only on those 11 breeds that were conditionally named core breeds and perform the appropriate IPM clustering for these 11 breeds based on the *EY*/*W* index (Fig. 3C; Supplementary data S4). Accordingly, inflection points were identified at *n*_1_ = 3.996, *n*_2_ = 5.311, and *n*_3_ = 8.171. If considering only 11 core breeds, it seems that a completely clear and plausible dependency graph could be obtained, starting with true ETB (without any “impurities” of non-relevant breeds) on the left side of the graph and ending with one real MTB on the right.

### SNP genotyping and admixture analysis

**Figure 4:**
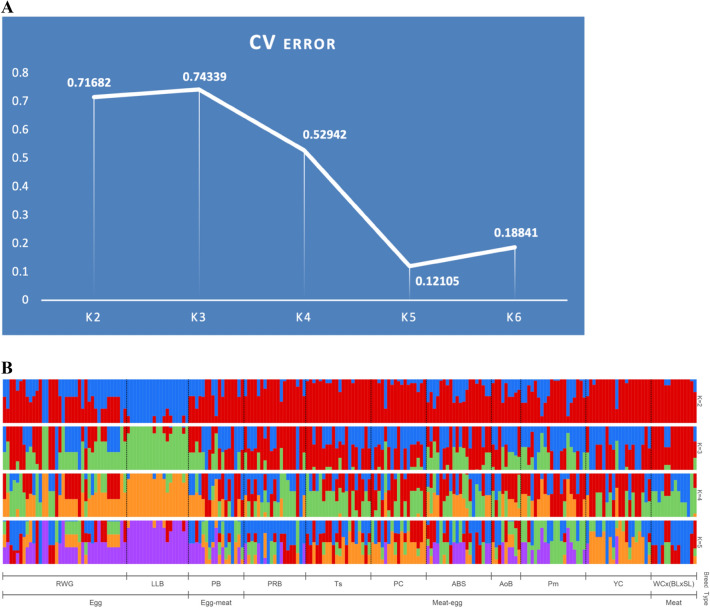
Population structure based on the genetic variation in the 11 core breeds genotyped for five SNP markers at the *NCAPG*-*LCORL* locus. (**A**) Elbow method-based analysis of cross-validation (CV) error values depending on number of ancestral populations (K). (**B**) Admixture bar plots generated by Bayesian clustering using the ADMIXTURE program. Each admixture plot represents a cluster structure of the studied breeds/breed types depending on number of ancestral populations (K), with the latter being optimal at K = 5. Core breeds: *RWG* Russian White, *LLB* Leghorn Light Brown, *PB* Pantsirevka Black, *PRB* Plymouth Rock Barred, *Ts* Tsarskoye Selo, *PC* Poltava Clay, *ABS* Australorp Black Speckled, *AoB* Australorp Black, *Pm* Pervomai, *YC* Yurlov Crower, *WC × (BL × SL)* White Cornish × (Brahma Light × Sussex Light).

Using genotypes in the 39 breeds for a total of five validated SNPs at the *NCAPG*-*LCORL* locus, LD analysis between SNP pairs (Supplementary Table S1) showed that some SNPs should have been omitted due to their complete heterozygosity in these breeds. In general, between the five SNP substitutions for all breeds, an average to weak LD level was observed. Full LD (*r*^2^ = 1) was found in six breeds such as CB (between GGaluGA265966 and Gga_rs14491028, and Gga_rs15619223 and Gga_rs14491017), Pu (between GGaluGA265969 and Gga_rs14491017), PS (between GGaluGA265966 and Gga_rs14491017), ZS (between rs14491017 and Gga_rs14491028), Ts (between GGaluGA265969 and Gga_rs14491017), and WC × (BL × SL) (between GGaluGA265969 and Gga_rs14491017). Admixture analysis for the 11 core breeds demonstrated the lowest error in the CV procedure at K = 5 (0.12105; Fig. 4A). Furthermore, a certain pattern of genetic differentiation was produced and visualized with the respective ADMIXTURE bar plots (Fig. 4B).

While K = 5 conformed to the most optimal and probable number of clusters (ancestral populations) (Fig. 4B), each breed type already had its own specific genetic structure at K = 3, although showing multiple instances of admixture and introgression from other breeds, except for LLB, a typical ETB, and the three-way crossbred population belonging to MTB. Particularly when K = 3, two ETB were predominantly characterized with a common ancestry of green color, one MTB had mostly a red-colored ancestry, and eight DPB (including both EMB and MEB) had a mixed ancestry of several colors. A similar pattern of clustering was observed at K = 4 and 5.

**Figure 5:**
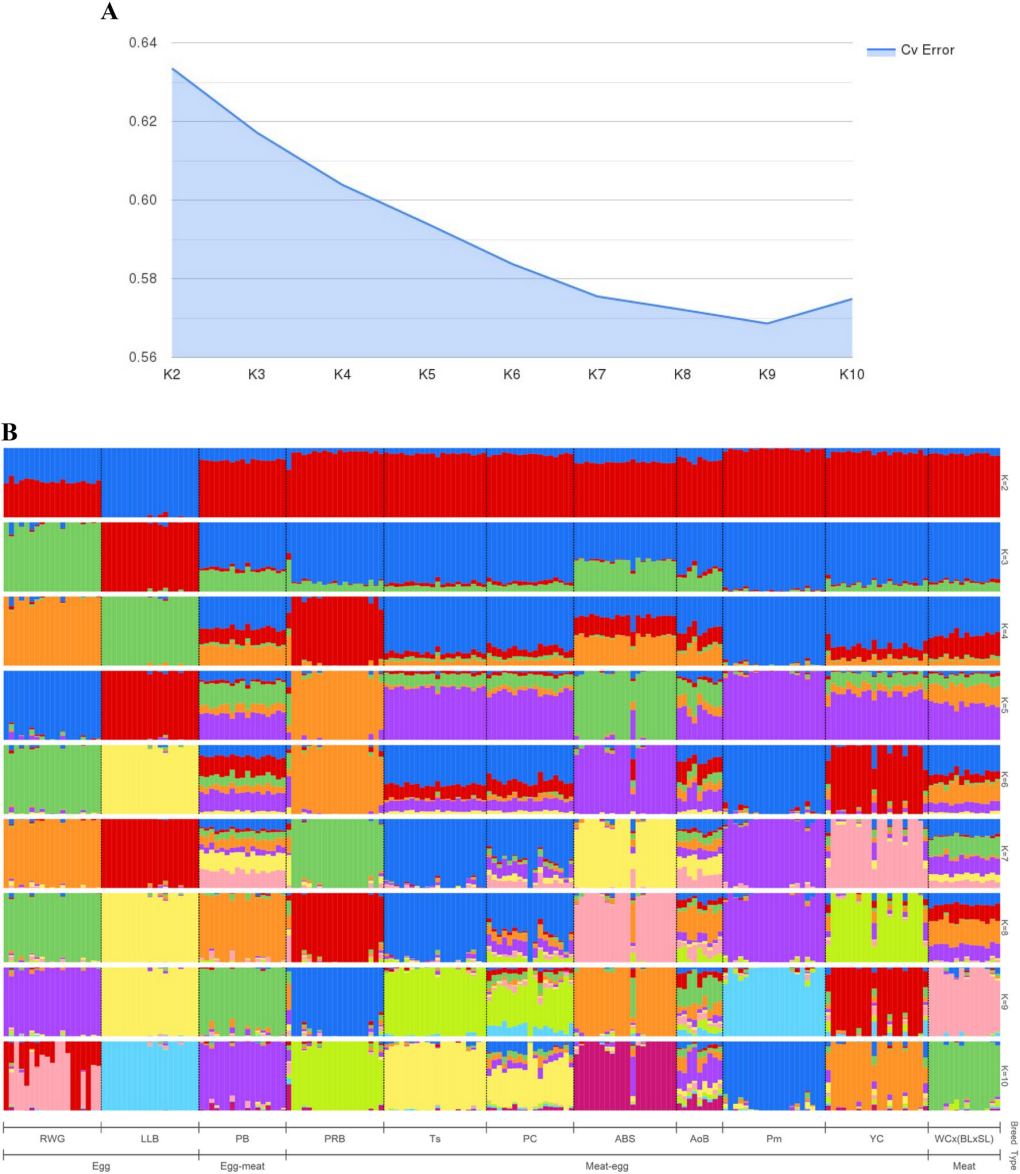
Population structure based on genome-wide genotypes in the 11 core breeds. (**A**) Elbow method-based analysis of cross-validation (CV) error values depending on number of ancestral populations (K). (**B**) Admixture bar plots generated by Bayesian clustering using the ADMIXTURE program. Each admixture plot represents a cluster structure of the studied breeds/breed types depending on number of ancestral populations (K), with the latter being optimal at K = 9. Core breeds: *RWG* Russian White, *LLB* Leghorn Light Brown, *PB* Pantsirevka Black, *PRB* Plymouth Rock Barred, *Ts* Tsarskoye Selo, *PC* Poltava Clay, *ABS* Australorp Black Speckled, *AoB* Australorp Black, *Pm* Pervomai, *YC* Yurlov Crower, *WC × (BL × SL)* White Cornish × (Brahma Light × Sussex Light).

Whole-genome SNP genotypes resulted in even clearer patterns of population structure for each core breed (Fig. 5B). The most optimal number of ancestral populations was achieved at K = 9 (Fig. 5B).

**Figure 6:**
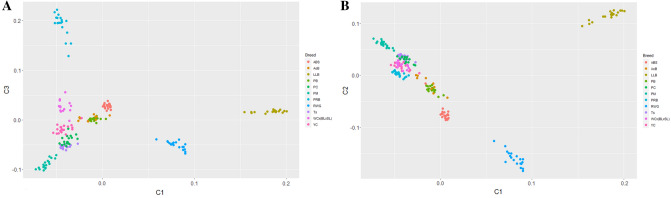
Results of PCA analysis of the 11 core chicken breeds. (**A**) First (C1) and third (C3) components. (**B**) First (C1) and second (C2) components.

**Figure 7:**
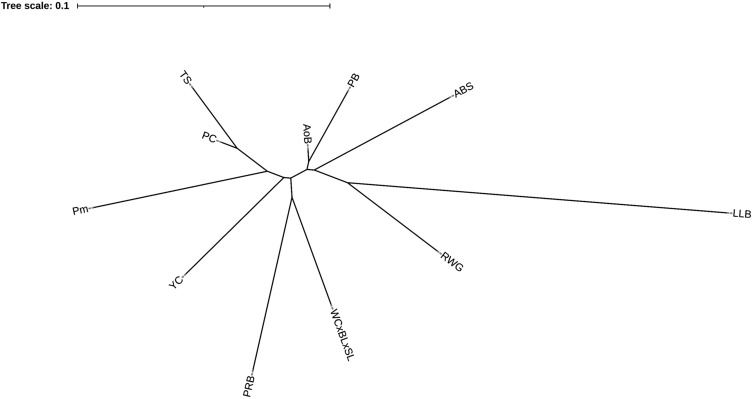
Phylogenetic tree based on pairwise *F*_ST_ genetic distances and built using the Neighbor-Net method.

Additionally, we conducted PCA analysis for the 11 core breeds using the same whole-genome genotype dataset (Fig. 6). Two ETB were remotely located from the rest breeds, with LLB being on the right side and RWG at the bottom of the PCA plot. Although all other breeds were located rather crowded on the plot, they were still quite separated from each other, especially WC × (BL × SL) of meat type and two MEB, YC and PRB. The PCA plot for the 39 breeds studied (see Supplementary Fig. S1) had five clusters: three for BTB (HSSD, SW–CB, BMF), one for two related breeds (BL–BB), and a conglomerate of other indistinctly separated breeds. In the phylogenetic tree (Fig. 7), there was an ETB cluster of LLB–RWG. The MTB WC × (BL × SL) formed a separate cluster joining further with the MEB PRB that is a maternal stock of another broiler cross. Other DPB occupied own clusters and single branches. Overall, the PCA and phylogenetic analyses provided an additional support to, and a proper comparison with, the admixture analysis outcome of the various breeds examined.

## Discussion

Assessing the diversity and genetic admixture of chicken breeds from local and world gene pools is an initial and important step to further the process of poultry breeding. It includes a powerful contemporary technique of genomic selection and the emerging field of phenome-wide association/mediation studies^42,43^. In the present study, we explored a number of clustering techniques to establish if the resulting breed groupings make biological sense. In particular, the data analysis, by implementing the integral performance coefficient *EY*/*W* (Fig. 3), allows us to evaluate the genetic potential of poultry performance.

Our *k*-means-based study (Supplementary data S1, Fig. 2) is consistent with the established notion that when there has been any prior group classification of cases, nonhierarchical clustering by the *k*-means method is a useful multivariate exploratory approach (e.g.,^25^). Its strength is that this technique focuses on categorizing groups in order to reduce variance within, and increase variance between, groups^44^.

While considering the previous three models, TCM, PCM and GCM^7^, we noticed that many breeds showed a different type (category) of clustering in different models. For example, a breed could conditionally be attributed to ETB using the first model, MEB according to the second and MTB according to the third. This may create some noise and bias in the analysis of 39 breeds, including their examination and clustering using the *k*-means (Supplementary data S1, Fig. 2) and new IPM approach (Fig. 3, Supplementary data S3 and S4).

We introduced a novel index such as the ratios of total egg mass yield to body weight of females (*EY*/*W*) and ranked them by descending order (Fig. 3). Logically, the higher the index, the higher egg-type properties of the layer/breed will be. There may be a rational kernel in this model; however, it is important to evaluate the adequacy of getting breeds into certain clusters using IPM. Herewith, one should bear in mind the following considerations. In particular, dwarf breeds (Bantams) have never been regarded as ETB. Apparently, DPB (both subtypes) are also not considered purely egg breeds. Therefore, in the first cluster (ETB, 1 to 12), only two breeds, the Russian White and the Leghorn Light Brown, are generally recognized as true egg breeds. The second, third and fourth clusters are also significantly mixed due to the presence of very different breeds in terms of performance (purpose of use) and genetic admixture. Thus, it can be assumed that the proposed model may not be much better than the four models described in Larkina et al.^7^. This, however, is not a flaw of new or old models; the examined breed composition itself is simply heterogenous from a genetic standpoint (see Fig. 4B), being often synthetic (composite) by origin and genetic structure. Nonetheless, we posit that the data obtained can be used, for instance, in the search for new mathematical models that allow *for* looking at the chicken breed gene pool from other and very interesting mathematical points of view. Perhaps, as a matter of discussion, it is worth assuming that the proposed model, in principle, is able to suggest (to a varying degree of certainty) the following four groups (Fig. 3): BTB, 1 to 3; a large group of ETB, DPB, FB and BTB (not included in the first group), 4 to 36; GB, 37 to 38; and MTB, 39. Therefore, these results also seem to us to be quite interesting for bearing in mind at developing mathematical models further for the clustering of chicken breeds.

Perhaps, the insufficient resolving power of IPM is explained by the fact that this model assumes the subdivision of breeds according to one specific index, albeit an integral one, i.e., the specific egg productivity relative to the body weight of laying hens. Most probably, BTB have large values of this proposed *EY*/*W* index mainly not due to its numerator, i.e., a supposedly high (or even greater than in true layers) level of egg performance, but due to its denominator, i.e., a significantly lower body weight (after all, they are dwarf, miniature chickens). It is difficult to imagine, of course, that BTB would be the preferred breeds for industrial scale egg production. This would makes no economic sense, and the commercial companies have not switched to dwarf layers. On the other hand, if BTB females, according to this index, lay eggs at the same (or even higher) level as classic ETB breeds, it might make sense to look at them in terms of including them in breeding programs aimed at developing breeds that produce more eggs.

In addition, one could think of some other integral indicator, for example, taking into account any external or other characteristic of chickens. For instance, Vakhrameev and Makarova^45^ listed different integral indices that describe exterior features, and one could take a closer look at these indices. In this case, clustering patterns may arise that do not follow generally accepted models. Rather, they are determined according to specific economically important traits and, consequently, to capabilities of a given breed to realize and improve its own egg performance-relevant genetic potential as a result of artificial selection.

It should also be noted whether dwarf breeds lay eggs of proper quality and nutritional value. When compared by egg weight, the differences between ETB and related breeds are fairly small. In general, among all breed groups (clusters) there is a certain uniformity in this trait. It can be assumed that this breakdown of breeds has occurred not in terms of how breeds of one or another selected performance trait should look like, but in terms of their degree of biological predisposition to egg production. Furthermore, this breed breakdown was obtained strictly in accordance with mathematical rules, which adds a certain attractiveness to it. In principle, in the first approximation, the breeds were, indeed, sorted according to the degree of their egg-type properties (left side of the graph in Fig. 3A) or meat-type properties (graph’s right side). Proceeding from the 39 chicken breeds (Fig. 3A) to 11 core breeds (Fig. 3C) resulted in more plausible clustering configuration pursuant to the respective *EY*/*W* function curve and inflection points. However, it is worth noting that a certain problem may arise in this case, which lies in the fact that by cutting the number of points, we can thereby smooth the curve (Supplementary data S4). Therefore, the inflection points do not become obvious, and it is not always possible to determine them. The proposed new method may seem rather controversial, so when aiming at developing a new, more suitable technique, one should plan to verify it in further studies using additional data. In any case, such search for an integral assessment of phenotypic traits can make an important contribution to developing genome- and phenome-related studies^43^ and strategies of germ plasm preservation^46,47^.

Finally, the admixture analysis results obtained are of special interest, since they were inferred from whole-genome SNP genotypes (Fig. 5) and those at the well-known *NCAPG*-*LCORL* genomic locus (Fig. 4) associated with productive traits in chickens. At K = 2, population structure of the 11 core breeds conformed to two basic ancestries as postulated by Moiseyeva et al.^6^, i.e., ETB (blue-colored in Figs. 4b and 5b) and MTB (red-colored). More mixed ancestries were revealed at K = 3, 4, 5 and so forth, although specific admixture patterns could generally be tracked for an individual breed and each of ETB, DPB and MTB groups. These admixture patterns for the 11 core breeds appeared to be more biologically meaningful than those previously described for the 39 breeds^7^ and did not contradict clustering configurations that resulted from using other methods tested here.

## Conclusions

The present study examined the importance of different methods for untangling complex clustering configurations among divergently selected chicken breeds representing a wide sampling of the world gene pool. We have demonstrated that different breeds can be classified (clustered) in one way or another depending on the chosen methods and (sub)models as well as on the degree of their genetic admixture. To this end, we have proposed a new integral indicator (i.e., *EY*/*W*), which links the main phenotypic traits in chickens, i.e., egg performance and body weight, as well as a mathematical model based on sorting breeds by inflection points. Future studies will use these findings to improve chicken breed clustering techniques as well as in genome- and phenome-wide association/mediation analyzes (e.g.,^1,42,43^) to elucidate cause-and-effect relationships between economically important characteristics, phenotypes, and SNP genotypes, including those at key associated loci, e.g., *NCAPG*-*LCORL* as we explored here.

## Supplementary Information

Supplementary Figure S1.

Supplementary Table S1.

Dataset S1.

Dataset S2.

Dataset S3.

Dataset S4.

## Data Availability

All data generated or analyzed during the present study are available from the corresponding author on reasonable request. The datasets supporting the conclusions of this article are included in the main manuscript and supplemental materials.

## Abbreviations

BTB: Bantam type breeds
CV: Cross-validation
DPB: Dual purpose breeds
EMB: Egg-meat breeds
ETB: Egg type breeds
EY: Egg mass yield
FB: Fancy breeds
GB: Game breeds
GCM: Genotypic clustering model
IPM: Inflection points model
K: Number of ancestral populations
MEB: Meat-egg breeds
MTB: Meat type breeds
PCM: Phenotypic clustering model
RRIFAGB: Russian Research Institute of Farm Animal Genetics and Breeding
*S*: Silhouette score
SNP: Single nucleotide polymorphism
SS: Sum of square distances
SSE: SS within groups
SSG: SS between groups
SST: Total SS
TCM: Traditional classification model
W: Body weight
