# Supplementary material for: Disentangling clustering configuration intricacies for divergently selected chicken breeds
Source: Sci Rep. 2023 Feb 27;13:3319. doi: 10.1038/s41598-023-28651-8 (PMC9971033; doi:10.1038/s41598-023-28651-8)
Supplement: Supplementary file 1 — Supplementary Figure S1. [file 41598_2023_28651_MOESM1_ESM.docx]

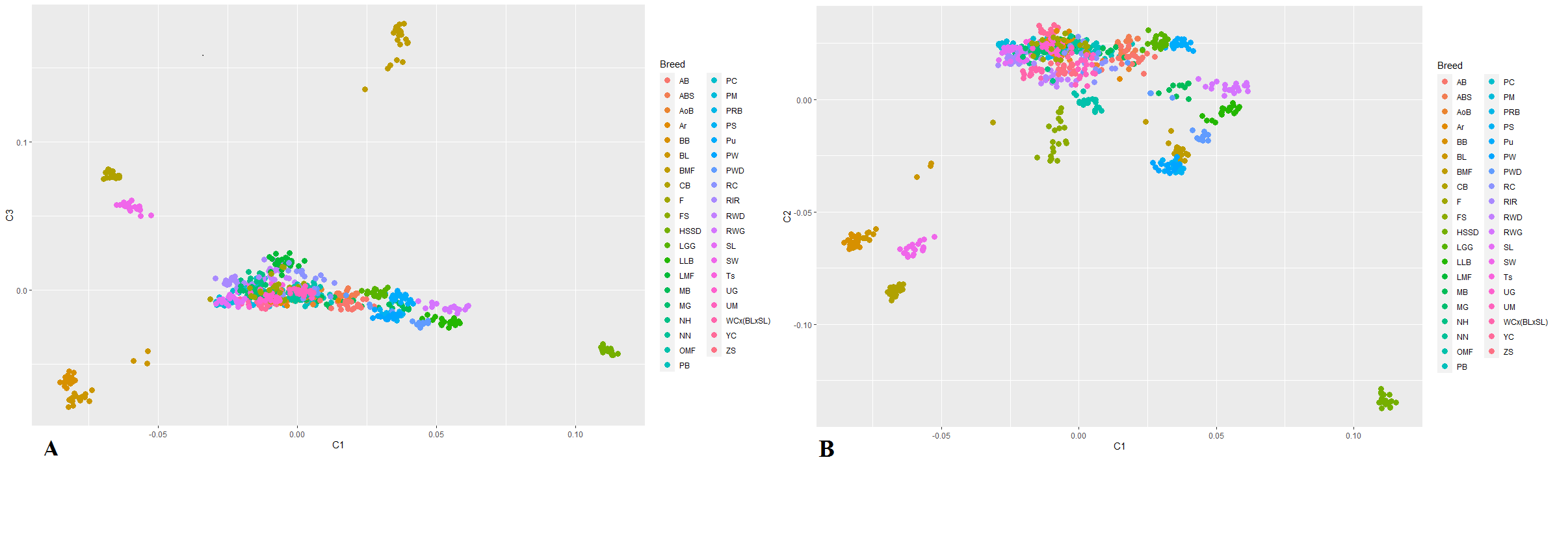


**Supplementary Fig. S1.** Results of PCA analysis of the 39 chicken breeds studied.

**A** First (C1) and third (C3) components. **B** First (C1) and second (C2) components.
