# Supplementary material for: Disentangling clustering configuration intricacies for divergently selected chicken breeds
Source: Sci Rep. 2023 Feb 27;13:3319. doi: 10.1038/s41598-023-28651-8 (PMC9971033; doi:10.1038/s41598-023-28651-8)
Supplement: Supplementary file 2 — Supplementary Table S1. [file 41598_2023_28651_MOESM2_ESM.docx]

**Supplementary Table S1.** Linkage disequilibrium (LD) analysis between SNP pairs at the *NCAPG-LCORL* locus in the 39 breeds studied.

| **Chromosome** | **Position** | **SNP** | **Position** | **SNP** | ***r*^2^** |
| --- | --- | --- | --- | --- | --- |
| Cochin Bantam / CB | | | | | |
| 4 | 78624706 | GGaluGA265966 | 78664025 | GGaluGA265969 | 0.57478 |
|  | 78624706 | GGaluGA265966 | 78673906 | Gga_rs15619223 | 0.30622 |
|  | 78624706 | GGaluGA265966 | 78755454 | Gga_rs14491017 | 0.30622 |
|  | 78624706 | GGaluGA265966 | 78775258 | Gga_rs14491028 | **1** |
|  | 78664025 | GGaluGA265969 | 78775258 | Gga_rs14491028 | 0.57478 |
|  | 78673906 | Gga_rs15619223 | 78755454 | Gga_rs14491017 | **1** |
|  | 78673906 | Gga_rs15619223 | 78775258 | Gga_rs14491028 | 0.30622 |
|  | 78755454 | Gga_rs14491017 | 78775258 | Gga_rs14491028 | 0.30622 |
| Red White-tailed Dwarf / RWD | | | | | |
| 4 | 78624706 | GGaluGA265966 | 78673906 | Gga_rs15619223 | 0.460925 |
|  | 78624706 | GGaluGA265966 | 78755454 | Gga_rs14491017 | 0.444444 |
|  | 78673906 | Gga_rs15619223 | 78755454 | Gga_rs14491017 | 0.460925 |
|  | 78673906 | Gga_rs15619223 | 78775258 | Gga_rs14491028 | 0.460925 |
| Bantam Mille Fleur / BMF | | | | | |
| 4 | 78673906 | Gga_rs15619223 | 78775258 | Gga_rs14491028 | 0.889039 |
| Russian White / RWG | | | | | |
| 4 | 78624706 | GGaluGA265966 | 78664025 | GGaluGA265969 | 0.86033 |
|  | 78624706 | GGaluGA265966 | 78673906 | Gga_rs15619223 | 0.35041 |
|  | 78624706 | GGaluGA265966 | 78755454 | Gga_rs14491017 | 0.313338 |
|  | 78624706 | GGaluGA265966 | 78775258 | Gga_rs14491028 | 0.855003 |
|  | 78664025 | GGaluGA265969 | 78673906 | Gga_rs15619223 | 0.339286 |
|  | 78664025 | GGaluGA265969 | 78755454 | Gga_rs14491017 | 0.429383 |
|  | 78664025 | GGaluGA265969 | 78775258 | Gga_rs14491028 | 0.740011 |
|  | 78673906 | Gga_rs15619223 | 78775258 | Gga_rs14491028 | 0.368534 |
|  | 78755454 | Gga_rs14491017 | 78775258 | Gga_rs14491028 | 0.216399 |
| Pushkin / Pu | | | | | |
| 4 | 78664025 | GGaluGA265969 | 78755454 | Gga_rs14491017 | **1** |
|  | 78664025 | GGaluGA265969 | 78775258 | Gga_rs14491028 | 0.462413 |
|  | 78755454 | Gga_rs14491017 | 78775258 | Gga_rs14491028 | 0.462413 |
| New Hampshire / NH | | | | | |
| 4 | 78624706 | GGaluGA265966 | 78755454 | Gga_rs14491017 | 0.390693 |
|  | 78624706 | GGaluGA265966 | 78775258 | Gga_rs14491028 | 0.216182 |
|  | 78664025 | GGaluGA265969 | 78755454 | Gga_rs14491017 | 0.821646 |
|  | 78755454 | Gga_rs14491017 | 78775258 | Gga_rs14491028 | 0.242424 |
| Hamburg Silver Spangled / HSSD | | | | | |
| 4 | 78624706 | GGaluGA265966 | 78673906 | Gga_rs15619223 | 0.382669 |
|  | 78624706 | GGaluGA265966 | 78775258 | Gga_rs14491028 | 0.363441 |
|  | 78673906 | Gga_rs15619223 | 78775258 | Gga_rs14491028 | 0.529412 |
| Dwarf Leningrad Mille Fleur / LMF | | | | | |
| 4 | 78624706 | GGaluGA265966 | 78755454 | Gga_rs14491017 | 0.516379 |
|  | 78624706 | GGaluGA265966 | 78775258 | Gga_rs14491028 | 0.370553 |
|  | 78664025 | GGaluGA265969 | 78673906 | Gga_rs15619223 | 0.315 |
|  | 78664025 | GGaluGA265969 | 78755454 | Gga_rs14491017 | 0.239726 |
|  | 78664025 | GGaluGA265969 | 78775258 | Gga_rs14491028 | 0.203636 |
|  | 78755454 | Gga_rs14491017 | 78775258 | Gga_rs14491028 | 0.739788 |
| Leghorn Light Brown / LLB | | | | | |
| 4 | 78673906 | Gga_rs15619223 | 78755454 | Gga_rs14491017 | 0.296296 |
| Leningrad Golden-and-gray / LGG | | | | | |
| 4 | 78624706 | GGaluGA265966 | 78673906 | Gga_rs15619223 | 0.409929 |
|  | 78624706 | GGaluGA265966 | 78775258 | Gga_rs14491028 | 0.795168 |
|  | 78664025 | GGaluGA265969 | 78755454 | Gga_rs14491017 | 0.655321 |
|  | 78673906 | Gga_rs15619223 | 78775258 | Gga_rs14491028 | 0.457627 |
| Aurora Blue / AB | | | | | |
| 4 | 78624706 | GGaluGA265966 | 78664025 | GGaluGA265969 | 0.469119 |
|  | 78624706 | GGaluGA265966 | 78673906 | Gga_rs15619223 | 0.482548 |
|  | 78624706 | GGaluGA265966 | 78775258 | Gga_rs14491028 | 0.698998 |
|  | 78664025 | GGaluGA265969 | 78755454 | Gga_rs14491017 | 0.398172 |
|  | 78664025 | GGaluGA265969 | 78775258 | Gga_rs14491028 | 0.655321 |
|  | 78673906 | Gga_rs15619223 | 78775258 | Gga_rs14491028 | 0.354582 |
| Amrock /Ar | | | | | |
| 4 | 78624706 | GGaluGA265966 | 78673906 | Gga_rs15619223 | 0.238095 |
|  | 78664025 | GGaluGA265969 | 78755454 | Gga_rs14491017 | 0.495197 |
|  | 78664025 | GGaluGA265969 | 78775258 | Gga_rs14491028 | 0.269753 |
|  | 78673906 | Gga_rs15619223 | 78775258 | Gga_rs14491028 | 0.214286 |
|  | 78755454 | Gga_rs14491017 | 78775258 | Gga_rs14491028 | 0.432169 |
| Rhode Island Red / RIR | | | | | |
| 4 | 78624706 | GGaluGA265966 | 78775258 | Gga_rs14491028 | 0.21039 |
|  | 78664025 | GGaluGA265969 | 78673906 | Gga_rs15619223 | 0.521739 |
|  | 78755454 | Gga_rs14491017 | 78775258 | Gga_rs14491028 | 0.625 |
| Pavlov Spangled / PS | | | | | |
| 4 | 78624706 | GGaluGA265966 | 78755454 | Gga_rs14491017 | **1** |
|  | 78673906 | Gga_rs15619223 | 78775258 | Gga_rs14491028 | 0.829401 |
| Poland White-crested Black / PWB | | | | | |
| 4 | 78624706 | GGaluGA265966 | 78673906 | Gga_rs15619223 | 0.460925 |
|  | 78624706 | GGaluGA265966 | 78755454 | Gga_rs14491017 | 0.444444 |
|  | 78673906 | Gga_rs15619223 | 78755454 | Gga_rs14491017 | 0.460925 |
|  | 78673906 | Gga_rs15619223 | 78775258 | Gga_rs14491028 | 0.460925 |
| Pantsirevka Black / PB | | | | | |
| 4 | 78624706 | GGaluGA265966 | 78664025 | GGaluGA265969 | 0.373752 |
|  | 78624706 | GGaluGA265966 | 78673906 | Gga_rs15619223 | 0.641293 |
|  | 78624706 | GGaluGA265966 | 78775258 | Gga_rs14491028 | 0.49 |
|  | 78664025 | GGaluGA265969 | 78673906 | Gga_rs15619223 | 0.357647 |
|  | 78664025 | GGaluGA265969 | 78755454 | Gga_rs14491017 | 0.289312 |
|  | 78673906 | Gga_rs15619223 | 78775258 | Gga_rs14491028 | 0.346154 |
| Russian Crested / RC | | | | | |
| 4 | 78624706 | GGaluGA265966 | 78673906 | Gga_rs15619223 | 0.225469 |
|  | 78624706 | GGaluGA265966 | 78755454 | Gga_rs14491017 | 0.571004 |
|  | 78624706 | GGaluGA265966 | 78775258 | Gga_rs14491028 | 0.471658 |
|  | 78673906 | Gga_rs15619223 | 78755454 | Gga_rs14491017 | 0.285948 |
|  | 78673906 | Gga_rs15619223 | 78775258 | Gga_rs14491028 | 0.329412 |
|  | 78755454 | Gga_rs14491017 | 78775258 | Gga_rs14491028 | 0.358175 |
| Frizzle / F | | | | | |
| 4 | 78624706 | GGaluGA265966 | 78664025 | GGaluGA265969 | 0.448019 |
|  | 78624706 | GGaluGA265966 | 78755454 | Gga_rs14491017 | 0.365882 |
|  | 78624706 | GGaluGA265966 | 78775258 | Gga_rs14491028 | 0.589744 |
|  | 78664025 | GGaluGA265969 | 78673906 | Gga_rs15619223 | 0.272727 |
|  | 78664025 | GGaluGA265969 | 78755454 | Gga_rs14491017 | 0.6978 |
|  | 78755454 | Gga_rs14491017 | 78775258 | Gga_rs14491028 | 0.286877 |
| Plymouth Rock Barred / PRB | | | | | |
| 4 | 78664025 | GGaluGA265969 | 78755454 | Gga_rs14491017 | 0.340643 |
|  | 78664025 | GGaluGA265969 | 78775258 | Gga_rs14491028 | 0.404447 |
|  | 78755454 | Gga_rs14491017 | 78775258 | Gga_rs14491028 | 0.254028 |
| Zagorsk Salmon / ZS | | | | | |
| 4 | 78624706 | GGaluGA265966 | 78673906 | Gga_rs15619223 | 0.309463 |
|  | 78624706 | GGaluGA265966 | 78755454 | Gga_rs14491017 | 0.240561 |
|  | 78624706 | GGaluGA265966 | 78775258 | Gga_rs14491028 | 0.240561 |
|  | 78664025 | GGaluGA265969 | 78755454 | Gga_rs14491017 | 0.892713 |
|  | 78673906 | Gga_rs15619223 | 78755454 | Gga_rs14491017 | 0.26161 |
|  | 78673906 | Gga_rs15619223 | 78775258 | Gga_rs14491028 | 0.26161 |
|  | 78755454 | Gga_rs14491017 | 78775258 | Gga_rs14491028 | **1** |
| Tsarskoye Selo / Ts | | | | | |
| 4 | 78664025 | GGaluGA265969 | 78755454 | Gga_rs14491017 | **1** |
|  | 78664025 | GGaluGA265969 | 78775258 | Gga_rs14491028 | 0.214463 |
|  | 78755454 | Gga_rs14491017 | 78775258 | Gga_rs14491028 | 0.214463 |
| Naked Neck / NN | | | | | |
| 4 | 78624706 | GGaluGA265966 | 78673906 | Gga_rs15619223 | 0.408163 |
|  | 78624706 | GGaluGA265966 | 78775258 | Gga_rs14491028 | 0.21327 |
|  | 78673906 | Gga_rs15619223 | 78755454 | Gga_rs14491017 | 0.396876 |
|  | 78673906 | Gga_rs15619223 | 78775258 | Gga_rs14491028 | 0.666312 |
|  | 78755454 | Gga_rs14491017 | 78775258 | Gga_rs14491028 | 0.619794 |
| Sussex Light / SL | | | | | |
| 4 | 78624706 | GGaluGA265966 | 78664025 | GGaluGA265969 | 0.228702 |
|  | 78664025 | GGaluGA265969 | 78755454 | Gga_rs14491017 | 0.722103 |
|  | 78664025 | GGaluGA265969 | 78775258 | Gga_rs14491028 | 0.437552 |
|  | 78755454 | Gga_rs14491017 | 78775258 | Gga_rs14491028 | 0.585511 |
| Poltava Clay / PC | | | | | |
| 4 | 78624706 | GGaluGA265966 | 78775258 | Gga_rs14491028 | 0.286107 |
|  | 78664025 | GGaluGA265969 | 78673906 | Gga_rs15619223 | 0.227273 |
|  | 78664025 | GGaluGA265969 | 78755454 | Gga_rs14491017 | 0.856237 |
|  | 78673906 | Gga_rs15619223 | 78775258 | Gga_rs14491028 | 0.23023 |
| Silkie White / SW | | | | | |
| 4 | 78624706 | GGaluGA265966 | 78755454 | Gga_rs14491017 | 0.380952 |
|  | 78624706 | GGaluGA265966 | 78775258 | Gga_rs14491028 | 0.204007 |
|  | 78755454 | Gga_rs14491017 | 78775258 | Gga_rs14491028 | 0.257143 |
| Minorca Black / MB | | | | | |
| 4 | 78624706 | GGaluGA265966 | 78664025 | GGaluGA265969 | 0.390788 |
|  | 78664025 | GGaluGA265969 | 78755454 | Gga_rs14491017 | 0.253947 |
|  | 78673906 | Gga_rs15619223 | 78755454 | Gga_rs14491017 | 0.410526 |
| Brahma Light / BL | | | | | |
| 4 | 78624706 | GGaluGA265966 | 78673906 | Gga_rs15619223 | 0.247215 |
|  | 78624706 | GGaluGA265966 | 78755454 | Gga_rs14491017 | 0.202739 |
|  | 78664025 | GGaluGA265969 | 78775258 | Gga_rs14491028 | 0.404762 |
|  | 78673906 | Gga_rs15619223 | 78755454 | Gga_rs14491017 | 0.563165 |
|  | 78673906 | Gga_rs15619223 | 78775258 | Gga_rs14491028 | 0.318107 |
| Pavlov White / PW | | | | | |
| 4 | 78624706 | GGaluGA265966 | 78673906 | Gga_rs15619223 | 0.327237 |
|  | 78624706 | GGaluGA265966 | 78755454 | Gga_rs14491017 | 0.864865 |
|  | 78673906 | Gga_rs15619223 | 78755454 | Gga_rs14491017 | 0.310345 |
|  | 78673906 | Gga_rs15619223 | 78775258 | Gga_rs14491028 | 0.689655 |
| Australorp Black Speckled / ABS | | | | | |
| 4 | 78664025 | GGaluGA265969 | 78755454 | Gga_rs14491017 | 0.409968 |
|  | 78664025 | GGaluGA265969 | 78775258 | Gga_rs14491028 | 0.244604 |
|  | 78755454 | Gga_rs14491017 | 78775258 | Gga_rs14491028 | 0.244604 |
| Australorp Black, Pervomai / AoB, Pm | | | | | |
| 4 | 78664025 | GGaluGA265969 | 78755454 | Gga_rs14491017 | 0.446429 |
|  | 78673906 | Gga_rs15619223 | 78755454 | Gga_rs14491017 | 0.5 |
|  | 78755454 | Gga_rs14491017 | 78775258 | Gga_rs14491028 | 0.240385 |
| Brahma Buff / BB | | | | | |
| 4 | 78624706 | GGaluGA265966 | 78673906 | Gga_rs15619223 | 0.65812 |
|  | 78624706 | GGaluGA265966 | 78755454 | Gga_rs14491017 | 0.741298 |
|  | 78624706 | GGaluGA265966 | 78775258 | Gga_rs14491028 | 0.254449 |
|  | 78664025 | GGaluGA265969 | 78755454 | Gga_rs14491017 | 0.352518 |
|  | 78664025 | GGaluGA265969 | 78775258 | Gga_rs14491028 | 0.583333 |
|  | 78673906 | Gga_rs15619223 | 78755454 | Gga_rs14491017 | 0.544628 |
|  | 78755454 | Gga_rs14491017 | 78775258 | Gga_rs14491028 | 0.468996 |
| Faverolles Salmon / FS | | | | | |
| 4 | 78624706 | GGaluGA265966 | 78664025 | GGaluGA265969 | 0.209402 |
|  | 78624706 | GGaluGA265966 | 78673906 | Gga_rs15619223 | 0.893998 |
|  | 78624706 | GGaluGA265966 | 78755454 | Gga_rs14491017 | 0.372583 |
|  | 78624706 | GGaluGA265966 | 78775258 | Gga_rs14491028 | 0.760026 |
|  | 78673906 | Gga_rs15619223 | 78755454 | Gga_rs14491017 | 0.535595 |
|  | 78673906 | Gga_rs15619223 | 78775258 | Gga_rs14491028 | 0.893998 |
|  | 78755454 | Gga_rs14491017 | 78775258 | Gga_rs14491028 | 0.533018 |
| Orloff Mille Fleur / ОMF | | | | | |
| 4 | 78624706 | GGaluGA265966 | 78673906 | Gga_rs15619223 | 0.494663 |
|  | 78624706 | GGaluGA265966 | 78755454 | Gga_rs14491017 | 0.360855 |
|  | 78624706 | GGaluGA265966 | 78775258 | Gga_rs14491028 | 0.612802 |
|  | 78664025 | GGaluGA265969 | 78755454 | Gga_rs14491017 | 0.401338 |
|  | 78673906 | Gga_rs15619223 | 78775258 | Gga_rs14491028 | 0.245888 |
|  | 78755454 | Gga_rs14491017 | 78775258 | Gga_rs14491028 | 0.583911 |
| Ukrainian Muffed / UM | | | | | |
| 4 | 78624706 | GGaluGA265966 | 78673906 | Gga_rs15619223 | 0.360034 |
|  | 78624706 | GGaluGA265966 | 78755454 | Gga_rs14491017 | 0.590765 |
|  | 78624706 | GGaluGA265966 | 78775258 | Gga_rs14491028 | 0.788897 |
|  | 78664025 | GGaluGA265969 | 78755454 | Gga_rs14491017 | 0.499057 |
|  | 78664025 | GGaluGA265969 | 78775258 | Gga_rs14491028 | 0.209938 |
|  | 78673906 | Gga_rs15619223 | 78755454 | Gga_rs14491017 | 0.222674 |
|  | 78673906 | Gga_rs15619223 | 78775258 | Gga_rs14491028 | 0.219319 |
|  | 78755454 | Gga_rs14491017 | 78775258 | Gga_rs14491028 | 0.590765 |
| Yurlov Crower / YC | | | | | |
| 4 | 78664025 | GGaluGA265969 | 78755454 | Gga_rs14491017 | 0.806469 |
|  | 78673906 | Gga_rs15619223 | 78775258 | Gga_rs14491028 | 0.448685 |
| Moscow Game / MG | | | | | |
| 4 | 78624706 | GGaluGA265966 | 78775258 | Gga_rs14491028 | 0.251397 |
|  | 78664025 | GGaluGA265969 | 78755454 | Gga_rs14491017 | 0.688286 |
|  | 78664025 | GGaluGA265969 | 78775258 | Gga_rs14491028 | 0.217877 |
|  | 78673906 | Gga_rs15619223 | 78755454 | Gga_rs14491017 | 0.240196 |
|  | 78755454 | Gga_rs14491017 | 78775258 | Gga_rs14491028 | 0.241976 |
| Uzbek Game / UG | | | | | |
| 4 | 78624706 | GGaluGA265966 | 78775258 | Gga_rs14491028 | 0.202111 |
|  | 78664025 | GGaluGA265969 | 78755454 | Gga_rs14491017 | 0.877518 |
|  | 78664025 | GGaluGA265969 | 78775258 | Gga_rs14491028 | 0.29918 |
|  | 78755454 | Gga_rs14491017 | 78775258 | Gga_rs14491028 | 0.262536 |
| White Cornish × (Brahma Light × Sussex Light) / WC × (BL × SL) | | | | | |
| 4 | 78664025 | GGaluGA265969 | 78755454 | Gga_rs14491017 | **1** |
|  | 78664025 | GGaluGA265969 | 78775258 | Gga_rs14491028 | 0.65 |
|  | 78755454 | Gga_rs14491017 | 78775258 | Gga_rs14491028 | 0.54 |
