# Supplementary material for: Disentangling clustering configuration intricacies for divergently selected chicken breeds
Source: Sci Rep. 2023 Feb 27;13:3319. doi: 10.1038/s41598-023-28651-8 (PMC9971033; doi:10.1038/s41598-023-28651-8)

**Supplementary data S1: K-means clustering**

**Statistics Kingdom**

[Home](https://www.statskingdom.com/index.html) > [Basic stats](https://www.statskingdom.com/basic-stats.html) > Cluster analysis

Cluster analysis

K-means clustering

Generates cluster graph and elbow chart.

URL: https://www.statskingdom.com/cluster-analysis.html

________________________________________________________________________________

**1. Original TCM-1 clustering (as shown in Table below)**

| **Breed type (TCM)** | **Breed type code** | ***n**** | **Breed** | ***EY*/*W*** | **Cluster** | **Silhouette (*S*)**** |
| --- | --- | --- | --- | --- | --- | --- |
| **Egg type** | 1 | **4** | RWG | 5.83 | 1 | 0.352 |
| **Egg type** | 1 | **9** | LLB | 4.77 | 1 | 0.5014 |
| **Egg type** | 1 | **26** | MB | 3.56 | 1 | 0.1896 |
| **Meat type** | 2 | **2** | RWD | 8.57 | 6 | 1 |
| **Meat type** | 2 | **39** | WC × (BL × SL) | 1.66 | 0 | 1 |
| **Egg-meat type** | 3 | **5** | Pu | 5.29 | 5 | 0.5686 |
| **Egg-meat type** | 3 | **6** | NH | 5.13 | 5 | 0.6022 |
| **Egg-meat type** | 3 | **8** | LMF | 5.06 | 5 | 0.6086 |
| **Egg-meat type** | 3 | **10** | LGG | 4.62 | 5 | 0.579 |
| **Egg-meat type** | 3 | **13** | RIR | 4.43 | 5 | 0.5231 |
| **Egg-meat type** | 3 | **16** | PB | 4.16 | 5 | 0.3684 |
| **Egg-meat type** | 3 | **20** | ZS | 3.81 | 5 | 0.05243 |
| **Meat-egg type** | 4 | **11** | AB | 4.54 | 4 | 0.0407 |
| **Meat-egg type** | 4 | **12** | Ar | 4.46 | 4 | -0.04592 |
| **Meat-egg type** | 4 | **19** | PRB | 4.06 | 4 | 0.4224 |
| **Meat-egg type** | 4 | **21** | Ts | 3.75 | 4 | 0.7293 |
| **Meat-egg type** | 4 | **22** | NN | 3.74 | 4 | 0.7361 |
| **Meat-egg type** | 4 | **24** | PC | 3.72 | 4 | 0.7459 |
| **Meat-egg type** | 4 | **23** | SL | 3.72 | 4 | 0.7459 |
| **Meat-egg type** | 4 | **29** | ABS | 3.34 | 7 | 0.7248 |
| **Meat-egg type** | 4 | **30** | AoB | 3.31 | 7 | 0.7157 |
| **Meat-egg type** | 4 | **31** | Pm | 3.27 | 7 | 0.6957 |
| **Meat-egg type** | 4 | **33** | FS | 3.23 | 7 | 0.6674 |
| **Meat-egg type** | 4 | **36** | YC | 2.92 | 7 | 0.3732 |
| **Game** | 5 | **34** | OMF | 3.03 | 7 | 0.6293 |
| **Game** | 5 | **37** | MG | 2.73 | 7 | 0.7873 |
| **Game** | 5 | **38** | UG | 2.48 | 7 | 0.7244 |
| **Fancy** | 6 | **1** | CB | 10.06 | 3 | 0.1894 |
| **Fancy** | 6 | **3** | BMF | 6.60 | 3 | -0.211 |
| **Fancy** | 6 | **7** | HSSD | 5.10 | 2 | 0.4658 |
| **Fancy** | 6 | **14** | PS | 4.36 | 2 | 0.6273 |
| **Fancy** | 6 | **15** | PWB | 4.23 | 2 | 0.6531 |
| **Fancy** | 6 | **17** | RC | 4.12 | 2 | 0.6636 |
| **Fancy** | 6 | **18** | F | 4.07 | 2 | 0.6622 |
| **Fancy** | 6 | **25** | SW | 3.70 | 2 | 0.5959 |
| **Fancy** | 6 | **27** | BL | 3.50 | 2 | 0.5205 |
| **Fancy** | 6 | **28** | PW | 3.46 | 2 | 0.4973 |
| **Fancy** | 6 | **32** | BB | 3.24 | 2 | 0.3197 |
| **Fancy** | 6 | **35** | UM | 2.92 | 2 | -0.01586 |
| Mean *S* | | | | | | 0.5130 |
| SD (*S*) | | | | | | 0.2782 |

**n*, serial number based on descending sorting by mean *EY*/*W* values.

**The Silhouette measures how similar the object is to its cluster (-1: non-similar, 1: very similar).


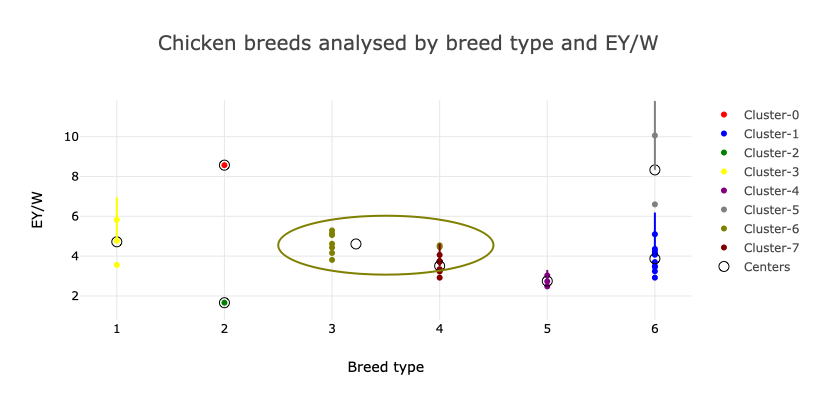


The data was divided into **8** clusters.
We chose the smallest k which explains at least 90% of the variance. (90.7373%)
Clusters: **3,3,3,0,2,6,6,6,6,6,6,6,6,6,7,7,7,7,7,7,7,7,7,7,4,4,4,5,5,1,1,1,1,1,1,1,1,1,1**. (See the cluster column above)
Explained variance ratio: **0.9074**.
SSE - The sum of squared of the distances from all the points to the centers.
SSE (Within): **16.7834**.
SSG (Between groups): 164.4101.
SST (Total): 181.1935.
SSE by group: 0, 3.6184, 0, 2.5802, 0.1517, 5.9858, 3.3932, 1.054.
Centers: [2,8.57], [6,3.87], [2,1.66], [1,4.72], [5,2.7467], [6,8.33], [3.2222,4.6111], [4,3.506].
Maximum iterations: 13.


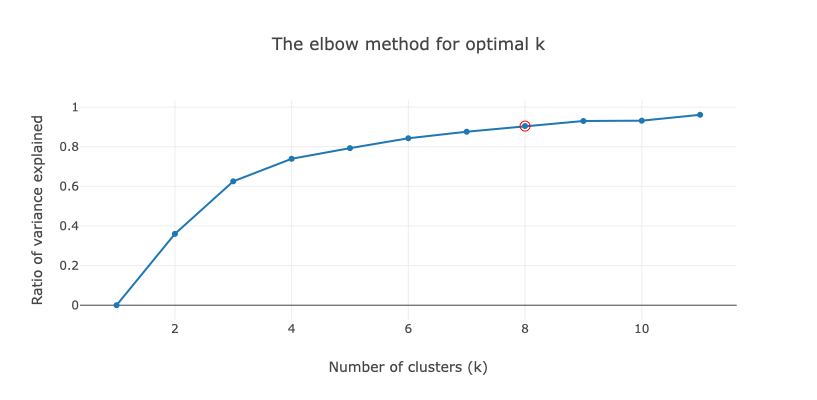


**2. Modified TCM-2 clustering based on descending sorting by mean *EY*/*W* values per breed type (as shown in tables below)**

| **Breed** | **Breed type code** | ***EY*/*W*** | **Mean by breed type** |
| --- | --- | --- | --- |
| RWG | 1 | 5.83 | 4.72 |
| LLB | 1 | 4.77 |  |
| MB | 1 | 3.56 |  |
| RWD | 2 | 8.57 | 5.115 |
| WC× | 2 | 1.66 |  |
| Pu | 3 | 5.29 | 4.64285714 |
| NH | 3 | 5.13 |  |
| LMF | 3 | 5.06 |  |
| LGG | 3 | 4.62 |  |
| RIR | 3 | 4.43 |  |
| PB | 3 | 4.16 |  |
| ZS | 3 | 3.81 |  |
| AB | 4 | 4.54 | 3.67166667 |
| Ar | 4 | 4.46 |  |
| PRB | 4 | 4.06 |  |
| Ts | 4 | 3.75 |  |
| NN | 4 | 3.74 |  |
| PC | 4 | 3.72 |  |
| SL | 4 | 3.72 |  |
| ABS | 4 | 3.34 |  |
| AoB | 4 | 3.31 |  |
| Pm | 4 | 3.27 |  |
| FS | 4 | 3.23 |  |
| YC | 4 | 2.92 |  |
| OMF | 5 | 3.03 | 2.74666667 |
| MG | 5 | 2.73 |  |
| UG | 5 | 2.48 |  |
| CB | 6 | 10.06 | 4.61333333 |
| BMF | 6 | 6.6 |  |
| HSSD | 6 | 5.1 |  |
| PS | 6 | 4.36 |  |
| PWB | 6 | 4.23 |  |
| RC | 6 | 4.12 |  |
| F | 6 | 4.07 |  |
| SW | 6 | 3.7 |  |
| BL | 6 | 3.5 |  |
| PW | 6 | 3.46 |  |
| BB | 6 | 3.24 |  |
| UM | 6 | 2.92 |  |

| **Breed type (TCM)** | **Breed type code** | ***n**** | **Breed** | ***EY*/*W*** | **Cluster** | **Silhouette (*S*)**** |
| --- | --- | --- | --- | --- | --- | --- |
| **Meat type** | 1 | **2** | RWD | 8.57 | 5 | 1 |
| **Meat type** | 1 | **39** | WC × (BL × SL) | 1.66 | 1 | 0.4026 |
| **Egg type** | 2 | **4** | RWG | 5.83 | 2 | 0.3368 |
| **Egg type** | 2 | **9** | LLB | 4.77 | 2 | 0.5109 |
| **Egg type** | 2 | **26** | MB | 3.56 | 2 | -0.2378 |
| **Egg-meat type** | 3 | **5** | Pu | 5.29 | 2 | 0.4305 |
| **Egg-meat type** | 3 | **6** | NH | 5.13 | 2 | 0.5173 |
| **Egg-meat type** | 3 | **8** | LMF | 5.06 | 2 | 0.5394 |
| **Egg-meat type** | 3 | **10** | LGG | 4.62 | 2 | 0.4768 |
| **Egg-meat type** | 3 | **13** | RIR | 4.43 | 2 | 0.3601 |
| **Egg-meat type** | 3 | **16** | PB | 4.16 | 2 | 0.09824 |
| **Egg-meat type** | 3 | **20** | ZS | 3.81 | 6 | 0.06999 |
| **Fancy** | 4 | **1** | CB | 10.06 | 0 | 1 |
| **Fancy** | 4 | **3** | BMF | 6.60 | 4 | 0.3088 |
| **Fancy** | 4 | **7** | HSSD | 5.10 | 6 | -0.09495 |
| **Fancy** | 4 | **14** | PS | 4.36 | 6 | 0.4132 |
| **Fancy** | 4 | **15** | PWB | 4.23 | 6 | 0.4726 |
| **Fancy** | 4 | **17** | RC | 4.12 | 6 | 0.5092 |
| **Fancy** | 4 | **18** | F | 4.07 | 6 | 0.518 |
| **Fancy** | 4 | **25** | SW | 3.70 | 6 | 0.5296 |
| **Fancy** | 4 | **27** | BL | 3.50 | 6 | 0.5092 |
| **Fancy** | 4 | **28** | PW | 3.46 | 6 | 0.4985 |
| **Fancy** | 4 | **32** | BB | 3.24 | 6 | 0.4077 |
| **Fancy** | 4 | **35** | UM | 2.92 | 6 | 0.2635 |
| **Meat-egg type** | 5 | **11** | AB | 4.54 | 7 | 0.3304 |
| **Meat-egg type** | 5 | **12** | Ar | 4.46 | 7 | 0.3632 |
| **Meat-egg type** | 5 | **19** | PRB | 4.06 | 7 | 0.524 |
| **Meat-egg type** | 5 | **21** | Ts | 3.75 | 7 | 0.6514 |
| **Meat-egg type** | 5 | **22** | NN | 3.74 | 7 | 0.6545 |
| **Meat-egg type** | 5 | **24** | PC | 3.72 | 7 | 0.6576 |
| **Meat-egg type** | 5 | **23** | SL | 3.72 | 7 | 0.6576 |
| **Meat-egg type** | 5 | **29** | ABS | 3.34 | 7 | 0.596 |
| **Meat-egg type** | 5 | **30** | AoB | 3.31 | 7 | 0.5816 |
| **Meat-egg type** | 5 | **31** | Pm | 3.27 | 7 | 0.5559 |
| **Meat-egg type** | 5 | **33** | FS | 3.23 | 7 | 0.5234 |
| **Meat-egg type** | 5 | **36** | YC | 2.92 | 7 | 0.2106 |
| **Game** | 6 | **34** | OMF | 3.03 | 3 | 0.6604 |
| **Game** | 6 | **37** | MG | 2.73 | 3 | 0.8058 |
| **Game** | 6 | **38** | UG | 2.48 | 3 | 0.7477 |
| Mean *S* | | | | | | 0.4708 |
| SD (*S*) | | | | | | 0.2467 |

**n*, serial number based on descending sorting by mean *EY*/*W* values.

**The Silhouette measures how similar the object is to his cluster (-1: non-similar, 1: very similar).


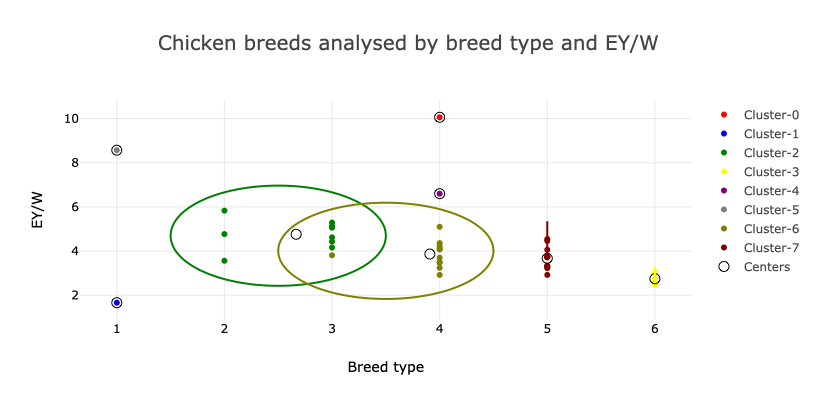


The data was divided into **8** clusters.
We chose the smallest k which explains at least 90% of the variance. (91.3793%)
Clusters: **5,1,2,2,2,2,2,2,2,2,2,6,0,4,6,6,6,6,6,6,6,6,6,6,7,7,7,7,7,7,7,7,7,7,7,7,3,3,3**. (See the cluster column above)
Explained variance ratio: **0.9138**.
SSE - The sum of squared of the distances from all the points to the centers.
SSE (Within): **12.9677**.
SSG (Between groups): 137.4566.
SST (Total): 150.4242.
SSE by group: 0, 0, 5.5813, 0.1517, 0, 0, 4.5308, 2.704.
Centers: [4,10.06], [1,1.66], [2.6667,4.7611], [6,2.7467], [4,6.6], [1,8.57], [3.9091,3.8645], [5,3.6717].
Maximum iterations: 17.


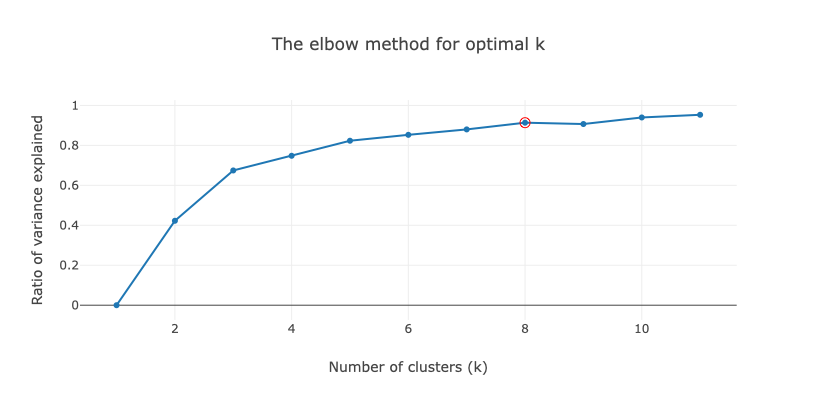


**3. Modified TCM-3 clustering based on descending sorting by greatest *EY*/*W* values per breed type (as shown in tables below)**

| **Breed** | **Breed type code** | ***EY*/*W*** |
| --- | --- | --- |
| CB | 1 | 10.06 |
| BMF | 1 | 6.6 |
| HSSD | 1 | 5.1 |
| PS | 1 | 4.36 |
| PWB | 1 | 4.23 |
| RC | 1 | 4.12 |
| F | 1 | 4.07 |
| SW | 1 | 3.7 |
| BL | 1 | 3.5 |
| PW | 1 | 3.46 |
| BB | 1 | 3.24 |
| UM | 1 | 2.92 |
| RWD | 2 | 8.57 |
| WC× | 2 | 1.66 |
| RWG | 3 | 5.83 |
| LLB | 3 | 4.77 |
| MB | 3 | 3.56 |
| Pu | 4 | 5.29 |
| NH | 4 | 5.13 |
| LMF | 4 | 5.06 |
| LGG | 4 | 4.62 |
| RIR | 4 | 4.43 |
| PB | 4 | 4.16 |
| ZS | 4 | 3.81 |
| AB | 5 | 4.54 |
| Ar | 5 | 4.46 |
| PRB | 5 | 4.06 |
| Ts | 5 | 3.75 |
| NN | 5 | 3.74 |
| PC | 5 | 3.72 |
| SL | 5 | 3.72 |
| ABS | 5 | 3.34 |
| AoB | 5 | 3.31 |
| Pm | 5 | 3.27 |
| FS | 5 | 3.23 |
| YC | 5 | 2.92 |
| OMF | 6 | 3.03 |
| MG | 6 | 2.73 |
| UG | 6 | 2.48 |

| **Breed type (TCM)** | **Breed type code** | ***n**** | **Breed** | ***EY*/*W*** | **Cluster** | **Silhouette (*S*)**** |
| --- | --- | --- | --- | --- | --- | --- |
| **Fancy** | 1 | **1** | CB | 10.06 | 4 | 0.5507 |
| **Fancy** | 1 | **3** | BMF | 6.60 | 4 | -0.03691 |
| **Fancy** | 1 | **7** | HSSD | 5.10 | 1 | 0.5123 |
| **Fancy** | 1 | **14** | PS | 4.36 | 1 | 0.7519 |
| **Fancy** | 1 | **15** | PWB | 4.23 | 1 | 0.7744 |
| **Fancy** | 1 | **17** | RC | 4.12 | 1 | 0.7841 |
| **Fancy** | 1 | **18** | F | 4.07 | 1 | 0.7845 |
| **Fancy** | 1 | **25** | SW | 3.70 | 1 | 0.7525 |
| **Fancy** | 1 | **27** | BL | 3.50 | 1 | 0.7103 |
| **Fancy** | 1 | **28** | PW | 3.46 | 1 | 0.6967 |
| **Fancy** | 1 | **32** | BB | 3.24 | 1 | 0.5876 |
| **Fancy** | 1 | **35** | UM | 2.92 | 3 | 0.3438 |
| **Meat type** | 2 | **2** | RWD | 8.57 | 4 | 0.5084 |
| **Meat type** | 2 | **39** | WC × (BL × SL) | 1.66 | 3 | 1 |
| **Egg type** | 3 | **4** | RWG | 5.83 | 2 | 0.5006 |
| **Egg type** | 3 | **9** | LLB | 4.77 | 2 | 0.5112 |
| **Egg-meat type** | 4 | **5** | Pu | 5.29 | 2 | 0.5955 |
| **Egg-meat type** | 4 | **6** | NH | 5.13 | 2 | 0.6154 |
| **Egg-meat type** | 4 | **8** | LMF | 5.06 | 2 | 0.6126 |
| **Egg-meat type** | 4 | **10** | LGG | 4.62 | 2 | 0.4898 |
| **Egg-meat type** | 4 | **13** | RIR | 4.43 | 2 | 0.3735 |
| **Egg-meat type** | 4 | **16** | PB | 4.16 | 2 | 0.1061 |
| **Egg-meat type** | 4 | **20** | ZS | 3.81 | 0 | 0.1177 |
| **Egg type** | 4 | **26** | MB | 3.56 | 0 | 0.2695 |
| **Meat-egg type** | 5 | **11** | AB | 4.54 | 0 | 0.2702 |
| **Meat-egg type** | 5 | **12** | Ar | 4.46 | 0 | 0.3305 |
| **Meat-egg type** | 5 | **19** | PRB | 4.06 | 0 | 0.5773 |
| **Meat-egg type** | 5 | **21** | Ts | 3.75 | 0 | 0.645 |
| **Meat-egg type** | 5 | **22** | NN | 3.74 | 0 | 0.6455 |
| **Meat-egg type** | 5 | **24** | PC | 3.72 | 0 | 0.6443 |
| **Meat-egg type** | 5 | **23** | SL | 3.72 | 0 | 0.6443 |
| **Meat-egg type** | 5 | **29** | ABS | 3.34 | 0 | 0.5193 |
| **Meat-egg type** | 5 | **30** | AoB | 3.31 | 0 | 0.5041 |
| **Meat-egg type** | 5 | **31** | Pm | 3.27 | 0 | 0.4781 |
| **Meat-egg type** | 5 | **33** | FS | 3.23 | 0 | 0.4463 |
| **Meat-egg type** | 5 | **36** | YC | 2.92 | 0 | 0.145 |
| **Game** | 6 | **34** | OMF | 3.03 | 5 | 0.6907 |
| **Game** | 6 | **37** | MG | 2.73 | 5 | 0.8204 |
| **Game** | 6 | **38** | UG | 2.48 | 5 | 0.7637 |
| Mean *S* | | | | | | 0.5138 |
| SD (*S*) | | | | | | 0.2253 |

**n*, serial number based on descending sorting by mean *EY*/*W* values.

**The Silhouette measures how similar the object is to his cluster (-1: non-similar, 1: very similar).


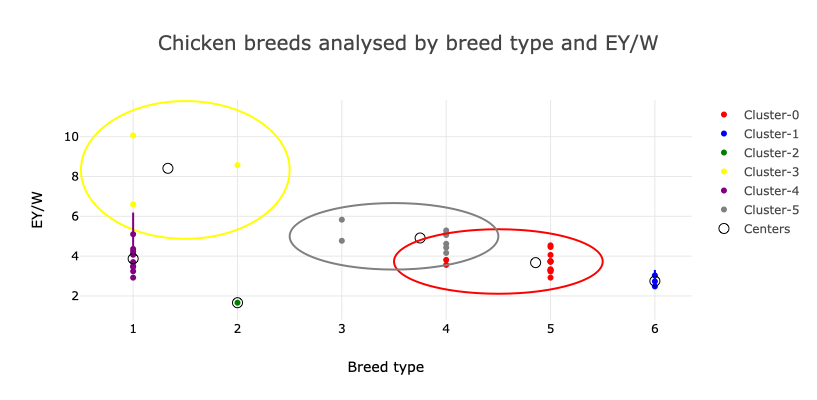


The data was divided into **6** clusters.
We chose the smallest k which explains at least 90% of the variance. (91.5225%)
Clusters: **3,3,4,4,4,4,4,4,4,4,4,4,3,2,5,5,5,5,5,5,5,5,0,0,0,0,0,0,0,0,0,0,0,0,0,0,1,1,1**. (See the cluster column above)
Explained variance ratio: **0.9152**.
SSE - The sum of squared of the distances from all the points to the centers.
SSE (Within): **18.369**.
SSG (Between groups): 198.3116.
SST (Total): 216.6806.
SSE by group: 4.4498, 0.1517, 0, 6.6909, 3.6184, 3.4583.
Centers: [4.8571,3.6736], [6,2.7467], [2,1.66], [1.3333,8.41], [1,3.87], [3.75,4.9112].
Maximum iterations: 11.


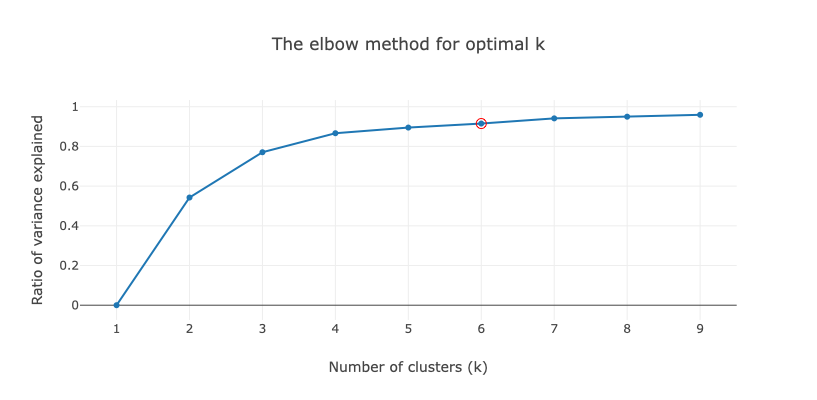


**4. Original PCM-1 clustering (as shown in Table below)**

| **Breed type (PCM)** | **Breed type code** | ***n**** | **Breed** | ***EY*/*W*** | **Cluster** | **Silhouette (*S*)**** |
| --- | --- | --- | --- | --- | --- | --- |
| **Bantam** | 1 | **1** | CB | 10.06 | 1 | 0.5582 |
| **Bantam** | 1 | **2** | RWD | 8.57 | 1 | 0.582 |
| **Bantam** | 1 | **3** | BMF | 6.60 | 1 | -0.1644 |
| **Egg type** | 2 | **4** | RWG | 5.83 | 5 | 0.2448 |
| **Meat-egg type** | 4 | **5** | Pu | 5.29 | 2 | 0.8907 |
| **Meat-egg type** | 4 | **6** | NH | 5.13 | 2 | 0.9307 |
| **Bantam** | 1 | **7** | HSSD | 5.10 | 5 | 0.4552 |
| **Meat-egg type** | 4 | **8** | LMF | 5.06 | 2 | 0.9068 |
| **Egg type** | 2 | **9** | LLB | 4.77 | 5 | 0.4095 |
| **Egg-meat type** | 3 | **10** | LGG | 4.62 | 6 | 0.2322 |
| **Egg type** | 2 | **11** | AB | 4.54 | 5 | 0.4095 |
| **Egg type** | 2 | **12** | Ar | 4.46 | 5 | 0.3929 |
| **Egg-meat type** | 3 | **13** | RIR | 4.43 | 6 | 0.4287 |
| **Fancy** | 7 | **14** | PS | 4.36 | 4 | 0.7913 |
| **Fancy** | 7 | **15** | PWB | 4.23 | 4 | 0.8092 |
| **Egg-meat type** | 3 | **16** | PB | 4.16 | 6 | 0.5806 |
| **Egg-meat type** | 3 | **17** | RC | 4.12 | 6 | 0.5854 |
| **Egg type** | 2 | **18** | F | 4.07 | 5 | 0.2206 |
| **Meat-egg type** | 4 | **19** | PRB | 4.06 | 0 | 0.3615 |
| **Meat-egg type** | 4 | **20** | ZS | 3.81 | 0 | 0.5511 |
| **Meat-egg type** | 4 | **21** | Ts | 3.75 | 0 | 0.5882 |
| **Egg type** | 2 | **22** | NN | 3.74 | 5 | 0.01397 |
| **Meat-egg type** | 4 | **24** | PC | 3.72 | 0 | 0.601 |
| **Egg-meat type** | 3 | **23** | SL | 3.72 | 6 | 0.5715 |
| **Bantam** | 1 | **25** | SW | 3.70 | 5 | 0.3174 |
| **Egg-meat type** | 3 | **26** | MB | 3.56 | 6 | 0.5409 |
| **Egg-meat type** | 3 | **27** | BL | 3.50 | 6 | 0.5173 |
| **Fancy** | 7 | **28** | PW | 3.46 | 4 | 0.5299 |
| **Meat-egg type** | 4 | **29** | ABS | 3.34 | 0 | 0.7004 |
| **Meat-egg type** | 4 | **30** | AoB | 3.31 | 0 | 0.7032 |
| **Meat-egg type** | 4 | **31** | Pm | 3.27 | 0 | 0.6999 |
| **Egg-meat type** | 3 | **32** | BB | 3.24 | 6 | 0.3707 |
| **Egg-meat type** | 3 | **33** | FS | 3.23 | 6 | 0.3635 |
| **Meat-egg type** | 4 | **34** | OMF | 3.03 | 0 | 0.6465 |
| **Meat-egg type** | 4 | **35** | UM | 2.92 | 0 | 0.6106 |
| **Meat-egg type** | 4 | **36** | YC | 2.92 | 0 | 0.6106 |
| **Game** | 6 | **37** | MG | 2.73 | 3 | 0.4808 |
| **Game** | 6 | **38** | UG | 2.48 | 3 | 0.5825 |
| **Meat type** | 5 | **39** | WC × (BL × SL) | 1.66 | 3 | 0.3198 |
| Mean *S* | | | | | | 0.5114 |
| SD (*S*) | | | | | | 0.2268 |

**n*, serial number based on descending sorting by mean *EY*/*W* values.

**The Silhouette measures how similar the object is to his cluster (-1: non-similar, 1: very similar).


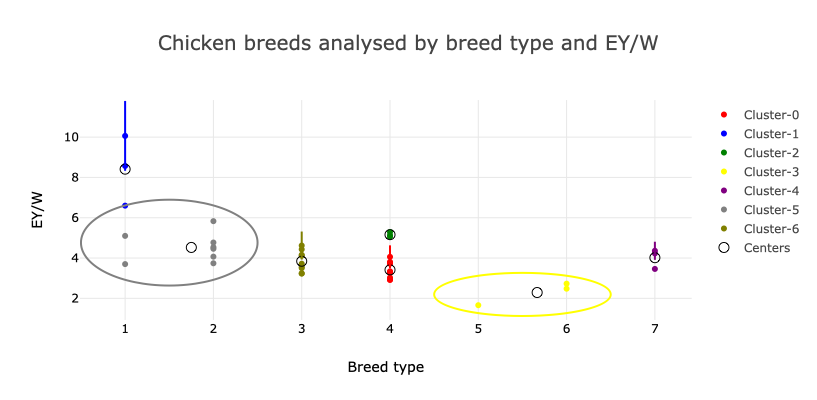


The data was divided into **7** clusters.
We chose the smallest k which explains at least 90% of the variance. (91.3884%)
Clusters: **1,1,1,5,2,2,5,2,5,6,5,5,6,4,4,6,6,5,0,0,0,5,0,6,5,6,6,4,0,0,0,6,6,0,0,0,3,3,3**. (See the cluster column above)
Explained variance ratio: **0.9139**.
SSE - The sum of squared of the distances from all the points to the centers.
SSE (Within): **16.4515**.
SSG (Between groups): 174.5881.
SST (Total): 191.0396.
SSE by group: 1.4532, 6.0242, 0.0278, 1.2933, 0.4733, 5.102, 2.0778.
Centers: [4,3.413], [1,8.41], [4,5.16], [5.6667,2.29], [7,4.0167], [1.75,4.5263], [3,3.8422].
Maximum iterations: 14.


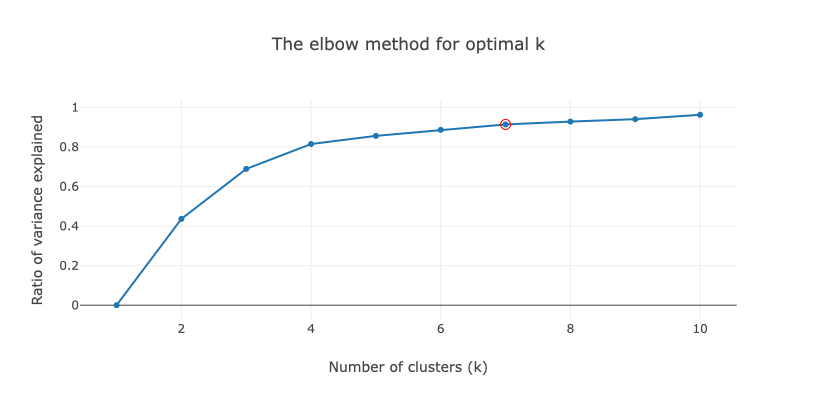


**5. Modified PCM-2 clustering based on descending sorting by mean *EY*/*W* values per breed type (as shown in tables below)**

| **Breed** | **Breed type code** | ***EY*/*W*** | **Mean by breed type** |
| --- | --- | --- | --- |
| CB | 1 | 10.06 | 6.81 |
| RWD | 1 | 8.57 |  |
| BMF | 1 | 6.60 |  |
| HSSD | 1 | 5.10 |  |
| SW | 1 | 3.70 |  |
| RWG | 2 | 5.83 | 4.57 |
| LLB | 2 | 4.77 |  |
| AB | 2 | 4.54 |  |
| Ar | 2 | 4.46 |  |
| F | 2 | 4.07 |  |
| NN | 2 | 3.74 |  |
| PS | 3 | 4.36 | 4.02 |
| PWB | 3 | 4.23 |  |
| PW | 3 | 3.46 |  |
| LGG | 4 | 4.62 | 3.84 |
| RIR | 4 | 4.43 |  |
| PB | 4 | 4.16 |  |
| RC | 4 | 4.12 |  |
| SL | 4 | 3.72 |  |
| MB | 4 | 3.56 |  |
| BL | 4 | 3.50 |  |
| BB | 4 | 3.24 |  |
| FS | 4 | 3.23 |  |
| Pu | 5 | 5.29 | 3.82 |
| NH | 5 | 5.13 |  |
| LMF | 5 | 5.06 |  |
| PRB | 5 | 4.06 |  |
| ZS | 5 | 3.81 |  |
| Ts | 5 | 3.75 |  |
| PC | 5 | 3.72 |  |
| ABS | 5 | 3.34 |  |
| AoB | 5 | 3.31 |  |
| Pm | 5 | 3.27 |  |
| ОMF | 5 | 3.03 |  |
| UM | 5 | 2.92 |  |
| YC | 5 | 2.92 |  |
| MG | 6 | 2.73 | 2.61 |
| UG | 6 | 2.48 |  |
| WC× | 7 | 1.66 |  |

| **Breed type (PCM)** | **Breed type code** | ***n**** | **Breed** | ***EY*/*W*** | **Cluster** | **Silhouette (*S*)**** |
| --- | --- | --- | --- | --- | --- | --- |
| **Bantam** | 1 | **1** | CB | 10.06 | 5 | 0.7222 |
| **Bantam** | 1 | **2** | RWD | 8.57 | 5 | 0.6177 |
| **Bantam** | 1 | **3** | BMF | 6.60 | 1 | 0.1644 |
| **Bantam** | 1 | **7** | HSSD | 5.10 | 1 | 0.5618 |
| **Bantam** | 1 | **25** | SW | 3.70 | 1 | 0.4303 |
| **Egg type** | 2 | **4** | RWG | 5.83 | 1 | 0.4142 |
| **Egg type** | 2 | **9** | LLB | 4.77 | 1 | 0.5063 |
| **Egg type** | 2 | **11** | AB | 4.54 | 1 | 0.5044 |
| **Egg type** | 2 | **12** | Ar | 4.46 | 1 | 0.4937 |
| **Egg type** | 2 | **18** | F | 4.07 | 1 | 0.3907 |
| **Egg type** | 2 | **22** | NN | 3.74 | 1 | 0.2665 |
| **Fancy** | 3 | **14** | PS | 4.36 | 4 | 0.3204 |
| **Fancy** | 3 | **15** | PWB | 4.23 | 4 | 0.3607 |
| **Fancy** | 3 | **28** | PW | 3.46 | 4 | 0.4387 |
| **Egg-meat type** | 4 | **10** | LGG | 4.62 | 4 | 0.1514 |
| **Egg-meat type** | 4 | **13** | RIR | 4.43 | 4 | 0.3342 |
| **Egg-meat type** | 4 | **16** | PB | 4.16 | 4 | 0.4661 |
| **Egg-meat type** | 4 | **17** | RC | 4.12 | 4 | 0.4664 |
| **Egg-meat type** | 4 | **23** | SL | 3.72 | 4 | 0.4141 |
| **Egg-meat type** | 4 | **26** | MB | 3.56 | 4 | 0.3719 |
| **Egg-meat type** | 4 | **27** | BL | 3.50 | 4 | 0.3473 |
| **Egg-meat type** | 4 | **32** | BB | 3.24 | 4 | 0.2115 |
| **Egg-meat type** | 4 | **33** | FS | 3.23 | 4 | 0.2052 |
| **Meat-egg type** | 5 | **5** | Pu | 5.29 | 3 | 0.8961 |
| **Meat-egg type** | 5 | **6** | NH | 5.13 | 3 | 0.933 |
| **Meat-egg type** | 5 | **8** | LMF | 5.06 | 3 | 0.9089 |
| **Meat-egg type** | 5 | **19** | PRB | 4.06 | 0 | 0.3465 |
| **Meat-egg type** | 5 | **20** | ZS | 3.81 | 0 | 0.63 |
| **Meat-egg type** | 5 | **21** | Ts | 3.75 | 0 | 0.6607 |
| **Meat-egg type** | 5 | **24** | PC | 3.72 | 0 | 0.6712 |
| **Meat-egg type** | 5 | **29** | ABS | 3.34 | 0 | 0.7499 |
| **Meat-egg type** | 5 | **30** | AoB | 3.31 | 0 | 0.7518 |
| **Meat-egg type** | 5 | **31** | Pm | 3.27 | 0 | 0.7483 |
| **Meat-egg type** | 5 | **34** | OMF | 3.03 | 0 | 0.6981 |
| **Meat-egg type** | 5 | **35** | UM | 2.92 | 0 | 0.6327 |
| **Meat-egg type** | 5 | **36** | YC | 2.92 | 0 | 0.6327 |
| **Game** | 6 | **37** | MG | 2.73 | 2 | 0.315 |
| **Game** | 6 | **38** | UG | 2.48 | 2 | 0.4474 |
| **Meat type** | 7 | **39** | WC × (BL × SL) | 1.66 | 2 | 0.4845 |
| Mean *S* | | | | | | 0.5043 |
| SD (*S*) | | | | | | 0.2044 |

**n*, serial number based on descending sorting by mean *EY*/*W* values.

**The Silhouette measures how similar the object is to his cluster (-1: non-similar, 1: very similar).


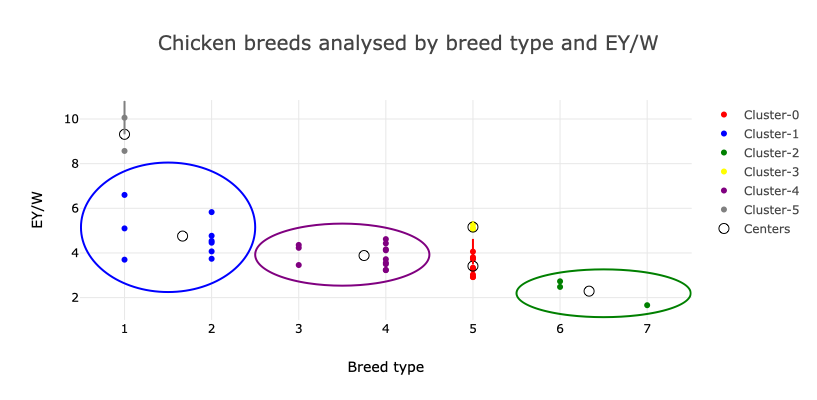


The data was divided into **6** clusters.
We chose the smallest k which explains at least 90% of the variance. (90.376%)
Clusters: **5,5,1,1,1,1,1,1,1,1,1,4,4,4,4,4,4,4,4,4,4,4,4,3,3,3,0,0,0,0,0,0,0,0,0,0,2,2,2**. (See the cluster column above)
Explained variance ratio: **0.9038**.
SSE - The sum of squared of the distances from all the points to the centers.
SSE (Within): **18.1784**.
SSG (Between groups): 170.7074.
SST (Total): 188.8858.
SSE by group: 1.4532, 9.4246, 1.2933, 0.0278, 4.8695, 1.1101.
Centers: [5,3.413], [1.6667,4.7567], [6.3333,2.29], [5,5.16], [3.75,3.8858], [1,9.315].
Maximum iterations: 13.


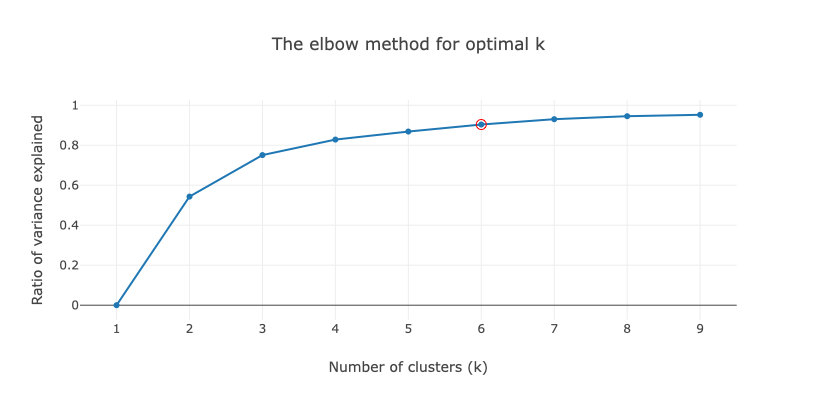


**6. Modified PCM-3 clustering based on descending sorting by greatest *EY*/*W* values per breed type (as shown in tables below)**

| **Breed** | **Breed type code** | ***EY*/*W*** |
| --- | --- | --- |
| CB | 1 | 10.06 |
| RWD | 1 | 8.57 |
| BMF | 1 | 6.60 |
| HSSD | 1 | 5.10 |
| SW | 1 | 3.70 |
| RWG | 2 | 5.83 |
| LLB | 2 | 4.77 |
| AB | 2 | 4.54 |
| Ar | 2 | 4.46 |
| F | 2 | 4.07 |
| NN | 2 | 3.74 |
| Pu | 3 | 5.29 |
| NH | 3 | 5.13 |
| LMF | 3 | 5.06 |
| PRB | 3 | 4.06 |
| ZS | 3 | 3.81 |
| Ts | 3 | 3.75 |
| PC | 3 | 3.72 |
| ABS | 3 | 3.34 |
| AoB | 3 | 3.31 |
| Pm | 3 | 3.27 |
| ОMF | 3 | 3.03 |
| UM | 3 | 2.92 |
| YC | 3 | 2.92 |
| LGG | 4 | 4.62 |
| RIR | 4 | 4.43 |
| PB | 4 | 4.16 |
| RC | 4 | 4.12 |
| SL | 4 | 3.72 |
| MB | 4 | 3.56 |
| BL | 4 | 3.50 |
| BB | 4 | 3.24 |
| FS | 4 | 3.23 |
| PS | 5 | 4.36 |
| PWB | 5 | 4.23 |
| PW | 5 | 3.46 |
| MG | 6 | 2.73 |
| UG | 6 | 2.48 |
| WC× | 7 | 1.66 |

| **Breed type (PCM)** | **Breed type code** | ***n**** | **Breed** | ***EY*/*W*** | **Cluster** | **Silhouette (*S*)**** |
| --- | --- | --- | --- | --- | --- | --- |
| **Bantam** | 1 | **1** | CB | 10.06 | 2 | 0.6499 |
| **Bantam** | 1 | **2** | RWD | 8.57 | 2 | 0.4651 |
| **Bantam** | 1 | **3** | BMF | 6.60 | 4 | 0.4399 |
| **Bantam** | 1 | **7** | HSSD | 5.10 | 4 | -0.03704 |
| **Bantam** | 1 | **25** | SW | 3.70 | 5 | 0.4071 |
| **Egg type** | 2 | **4** | RWG | 5.83 | 4 | -0.03495 |
| **Egg type** | 2 | **9** | LLB | 4.77 | 5 | 0.3065 |
| **Egg type** | 2 | **11** | AB | 4.54 | 5 | 0.5106 |
| **Egg type** | 2 | **12** | Ar | 4.46 | 5 | 0.5494 |
| **Egg type** | 2 | **18** | F | 4.07 | 5 | 0.5235 |
| **Egg type** | 2 | **22** | NN | 3.74 | 5 | 0.303 |
| **Egg-meat type** | 4 | **10** | LGG | 4.62 | 3 | 0.8788 |
| **Egg-meat type** | 4 | **13** | RIR | 4.43 | 3 | 0.9238 |
| **Egg-meat type** | 4 | **16** | PB | 4.16 | 3 | 0.8979 |
| **Egg-meat type** | 4 | **17** | RC | 4.12 | 1 | 0.3465 |
| **Egg-meat type** | 4 | **23** | SL | 3.72 | 1 | 0.6207 |
| **Egg-meat type** | 4 | **26** | MB | 3.56 | 1 | 0.6566 |
| **Egg-meat type** | 4 | **27** | BL | 3.50 | 1 | 0.6712 |
| **Egg-meat type** | 4 | **32** | BB | 3.24 | 1 | 0.7499 |
| **Egg-meat type** | 4 | **33** | FS | 3.23 | 1 | 0.7518 |
| **Meat-egg type** | 3 | **5** | Pu | 5.29 | 1 | 0.7483 |
| **Meat-egg type** | 3 | **6** | NH | 5.13 | 1 | 0.6981 |
| **Meat-egg type** | 3 | **8** | LMF | 5.06 | 1 | 0.6644 |
| **Meat-egg type** | 3 | **19** | PRB | 4.06 | 1 | 0.6644 |
| **Meat-egg type** | 3 | **20** | ZS | 3.81 | 6 | 0.1514 |
| **Meat-egg type** | 3 | **21** | Ts | 3.75 | 6 | 0.3342 |
| **Meat-egg type** | 3 | **24** | PC | 3.72 | 6 | 0.4661 |
| **Meat-egg type** | 3 | **29** | ABS | 3.34 | 6 | 0.4664 |
| **Meat-egg type** | 3 | **30** | AoB | 3.31 | 6 | 0.4141 |
| **Meat-egg type** | 3 | **31** | Pm | 3.27 | 6 | 0.3719 |
| **Meat-egg type** | 3 | **34** | OMF | 3.03 | 6 | 0.3473 |
| **Meat-egg type** | 3 | **35** | UM | 2.92 | 6 | 0.2115 |
| **Meat-egg type** | 3 | **36** | YC | 2.92 | 6 | 0.2052 |
| **Meat type** | 7 | **39** | WC × (BL × SL) | 1.66 | 6 | 0.4977 |
| **Fancy** | 5 | **14** | PS | 4.36 | 6 | 0.5272 |
| **Fancy** | 5 | **15** | PWB | 4.23 | 6 | 0.3806 |
| **Fancy** | 5 | **28** | PW | 3.46 | 0 | 0.6026 |
| **Game** | 6 | **37** | MG | 2.73 | 0 | 0.6647 |
| **Game** | 6 | **38** | UG | 2.48 | 0 | 0.6141 |
| Mean *S* | | | | | | 0.5028 |
| SD (*S*) | | | | | | 0.2281 |

**n*, serial number based on descending sorting by mean *EY*/*W* values.

**The Silhouette measures how similar the object is to his cluster (-1: non-similar, 1: very similar).


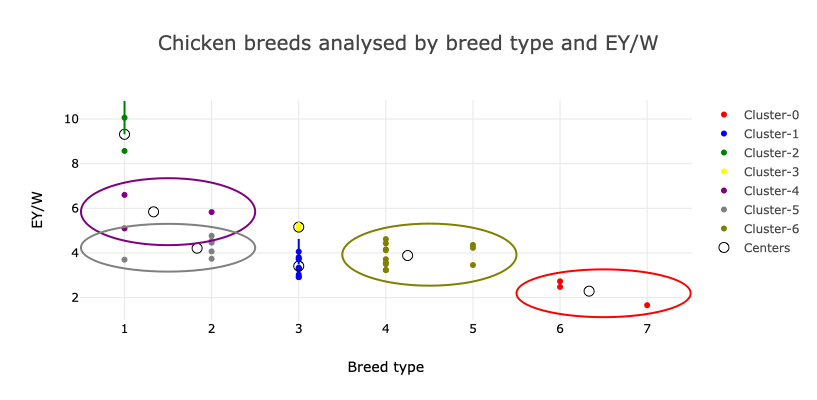


The data was divided into **7** clusters.
We chose the smallest k which explains at least 90% of the variance. (92.6564%)
Clusters: **2,2,4,4,5,4,5,5,5,5,5,3,3,3,1,1,1,1,1,1,1,1,1,1,6,6,6,6,6,6,6,6,6,6,6,6,0,0,0**. (See the cluster column above)
Explained variance ratio: **0.9266**.
SSE - The sum of squared of the distances from all the points to the centers.
SSE (Within): **12.3646**.
SSG (Between groups): 156.0083.
SST (Total): 168.373.
SSE by group: 1.2933, 1.4532, 1.1101, 0.0278, 1.7919, 1.8189, 4.8695.
Centers: [6.3333,2.29], [3,3.413], [1,9.315], [3,5.16], [1.3333,5.8433], [1.8333,4.2133], [4.25,3.8858].
Maximum iterations: 13.


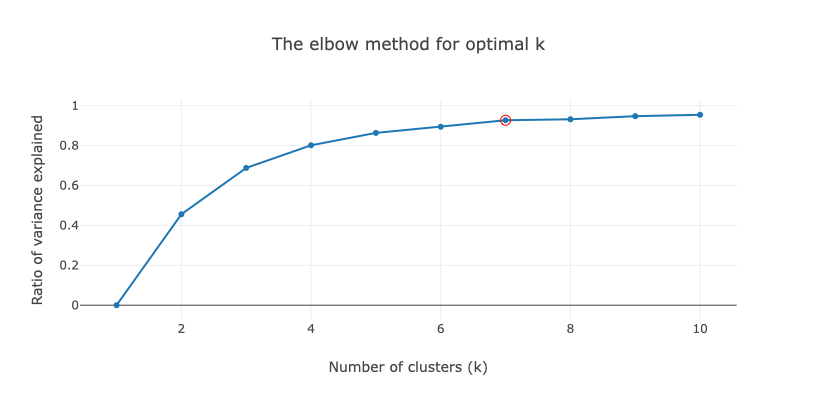


**7. Original GCM-1 clustering (as shown in Table below)**

| **Breed type (GCM1)** | **Breed type code** | ***n**** | **Breed** | ***EY*/*W*** | **Cluster** | **Silhouette (*S*)**** |
| --- | --- | --- | --- | --- | --- | --- |
| **Egg Ia type** | 1 | **3** | BMF | 6.60 | 3 | 0.463 |
| **Egg Ia type** | 1 | **7** | HSSD | 5.10 | 3 | 0.2968 |
| **Egg Ia type** | 1 | **9** | LLB | 4.77 | 1 | -0.02254 |
| **Egg Ia type** | 1 | **14** | PS | 4.36 | 1 | 0.239 |
| **Egg Ia type** | 1 | **15** | PWB | 4.23 | 1 | 0.3504 |
| **Egg Ia type** | 1 | **26** | MB | 3.56 | 1 | 0.5742 |
| **Egg Ia type** | 1 | **28** | PW | 3.46 | 1 | 0.5566 |
| **Egg Ib type** | 2 | **4** | RWG | 5.83 | 3 | 0.3701 |
| **Egg Ib type** | 2 | **13** | RIR | 4.43 | 1 | 0.2811 |
| **Egg Ib type** | 2 | **17** | RC | 4.12 | 1 | 0.337 |
| **Egg Ib type** | 2 | **22** | NN | 3.74 | 1 | 0.3383 |
| **Egg Ib type** | 2 | **27** | BL | 3.50 | 1 | 0.2902 |
| **Egg Ib type** | 2 | **33** | FS | 3.23 | 1 | 0.1894 |
| **Dual purpose IIa type** | 3 | **11** | AB | 4.54 | 0 | 0.3201 |
| **Dual purpose IIa type** | 3 | **12** | Ar | 4.46 | 0 | 0.3454 |
| **Dual purpose IIa type** | 3 | **16** | PB | 4.16 | 0 | 0.4182 |
| **Dual purpose IIa type** | 3 | **18** | F | 4.07 | 0 | 0.4323 |
| **Dual purpose IIa type** | 3 | **23** | SL | 3.72 | 0 | 0.4519 |
| **Dual purpose IIa type** | 3 | **29** | ABS | 3.34 | 0 | 0.4529 |
| **Dual purpose IIa type** | 3 | **31** | Pm | 3.27 | 0 | 0.4452 |
| **Dual purpose IIa type** | 3 | **34** | OMF | 3.03 | 0 | 0.3881 |
| **Dual purpose IIa type** | 3 | **36** | YC | 2.92 | 0 | 0.3509 |
| **Dual purpose IIa type** | 3 | **35** | UM | 2.92 | 0 | 0.3509 |
| **Dual purpose IIb type** | 4 | **8** | LMF | 5.06 | 5 | 0.1708 |
| **Dual purpose IIb type** | 4 | **19** | PRB | 4.06 | 4 | 0.4382 |
| **Dual purpose IIb type** | 4 | **21** | Ts | 3.75 | 4 | 0.6138 |
| **Dual purpose IIb type** | 4 | **24** | PC | 3.72 | 4 | 0.6196 |
| **Dual purpose IIb type** | 4 | **30** | AoB | 3.31 | 4 | 0.5437 |
| **Dual purpose IIb type** | 4 | **37** | MG | 2.73 | 4 | 0.1874 |
| **Meat type** | 5 | **1** | CB | 10.06 | 6 | 0.7278 |
| **Meat type** | 5 | **2** | RWD | 8.57 | 6 | 0.6267 |
| **Meat type** | 5 | **5** | Pu | 5.29 | 5 | 0.5194 |
| **Meat type** | 5 | **6** | NH | 5.13 | 5 | 0.5381 |
| **Meat type** | 5 | **10** | LGG | 4.62 | 5 | 0.4736 |
| **Meat type** | 5 | **20** | ZS | 3.81 | 2 | 0.05434 |
| **Meat type** | 5 | **25** | SW | 3.70 | 2 | -0.03612 |
| **Meat type** | 5 | **32** | BB | 3.24 | 2 | -0.03419 |
| **Meat type** | 5 | **38** | UG | 2.48 | 2 | 0.4669 |
| **Meat type** | 5 | **39** | WC × (BL × SL) | 1.66 | 2 | 0.434 |
| Mean *S* | | | | | | 0.3734 |
| SD (*S*) | | | | | | 0.1826 |

**n*, serial number based on descending sorting by mean *EY*/*W* values.

**The Silhouette measures how similar the object is to his cluster (-1: non-similar, 1: very similar).


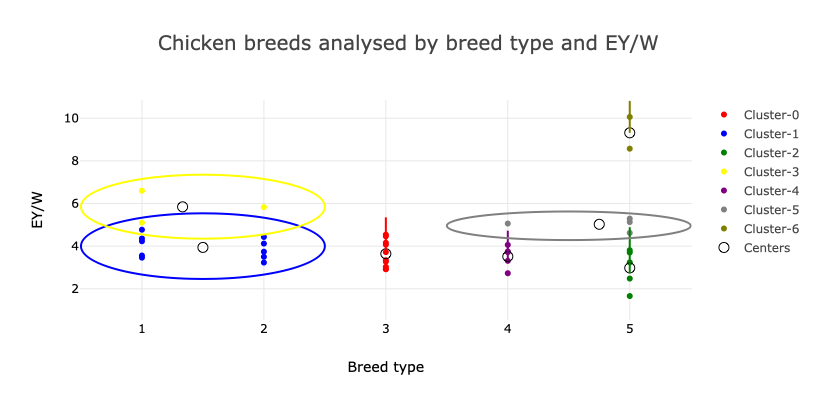


The data was divided into **7** clusters.
We chose the smallest k which explains at least 90% of the variance. (90.1307%)
Clusters: **3,3,1,1,1,1,1,3,1,1,1,1,1,0,0,0,0,0,0,0,0,0,0,5,4,4,4,4,4,6,6,5,5,5,2,2,2,2,2**. (See the cluster column above)
Explained variance ratio: **0.9013**.
SSE - The sum of squared of the distances from all the points to the centers.
SSE (Within): **16.6325**.
SSG (Between groups): 151.8943.
SST (Total): 168.5268.
SSE by group: 3.5798, 4.8344, 3.2673, 1.7919, 1.0525, 0.9965, 1.1101.
Centers: [3,3.643], [1.5,3.94], [5,2.978], [1.3333,5.8433], [4,3.514], [4.75,5.025], [5,9.315].
Maximum iterations: 11.


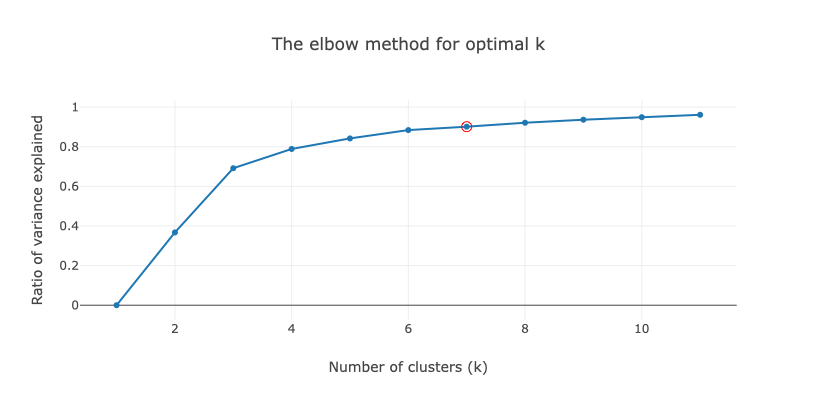


**8. Modified GCM-2 clustering based on descending sorting by mean *EY*/*W* values per breed type. This coincides with the Modified GCM-3 clustering based on descending sorting by greatest *EY*/*W* values per breed type (as shown in tables below)**

| **Breed** | **Breed type code** | ***EY*/*W*** | **Mean by breed type** |
| --- | --- | --- | --- |
| CB | 1 | 10.06 | 4.86 |
| RWD | 1 | 8.57 |  |
| Pu | 1 | 5.29 |  |
| NH | 1 | 5.13 |  |
| LGG | 1 | 4.62 |  |
| ZS | 1 | 3.81 |  |
| SW | 1 | 3.70 |  |
| BB | 1 | 3.24 |  |
| UG | 1 | 2.48 |  |
| WC× | 1 | 1.66 |  |
| BMF | 2 | 6.60 | 4.58 |
| HSSD | 2 | 5.10 |  |
| LLB | 2 | 4.77 |  |
| PS | 2 | 4.36 |  |
| PWB | 2 | 4.23 |  |
| MB | 2 | 3.56 |  |
| PW | 2 | 3.46 |  |
| RWG | 3 | 5.83 | 4.14 |
| RIR | 3 | 4.43 |  |
| RC | 3 | 4.12 |  |
| NN | 3 | 3.74 |  |
| BL | 3 | 3.50 |  |
| FS | 3 | 3.23 |  |
| LMF | 4 | 5.06 | 3.77 |
| PRB | 4 | 4.06 |  |
| Ts | 4 | 3.75 |  |
| PC | 4 | 3.72 |  |
| AoB | 4 | 3.31 |  |
| MG | 4 | 2.73 |  |
| AB | 5 | 4.54 | 3.64 |
| Ar | 5 | 4.46 |  |
| PB | 5 | 4.16 |  |
| F | 5 | 4.07 |  |
| SL | 5 | 3.72 |  |
| ABS | 5 | 3.34 |  |
| Pm | 5 | 3.27 |  |
| ОMF | 5 | 3.03 |  |
| UM | 5 | 2.92 |  |
| YC | 5 | 2.92 |  |

| **Breed type (GCM1)** | **Breed type code** | ***n**** | **Breed** | ***EY*/*W*** | **Cluster** | **Silhouette (*S*)**** |
| --- | --- | --- | --- | --- | --- | --- |
| **Egg Ia type** | 2 | **3** | BMF | 6.60 | 4 | 0.6817 |
| **Egg Ia type** | 2 | **7** | HSSD | 5.10 | 4 | 0.5465 |
| **Egg Ia type** | 2 | **9** | LLB | 4.77 | 2 | 0.335 |
| **Egg Ia type** | 2 | **14** | PS | 4.36 | 2 | 0.4262 |
| **Egg Ia type** | 2 | **15** | PWB | 4.23 | 2 | 0.5641 |
| **Egg Ia type** | 2 | **26** | MB | 3.56 | 2 | 0.3111 |
| **Egg Ia type** | 2 | **28** | PW | 3.46 | 2 | 0.1835 |
| **Egg Ib type** | 3 | **4** | RWG | 5.83 | 0 | 0.07874 |
| **Egg Ib type** | 3 | **13** | RIR | 4.43 | 0 | 0.6108 |
| **Egg Ib type** | 3 | **17** | RC | 4.12 | 0 | 0.5605 |
| **Egg Ib type** | 3 | **22** | NN | 3.74 | 3 | 0.3445 |
| **Egg Ib type** | 3 | **27** | BL | 3.50 | 3 | 0.2109 |
| **Egg Ib type** | 3 | **33** | FS | 3.23 | 3 | 0.003823 |
| **Dual purpose IIa type** | 5 | **11** | AB | 4.54 | 1 | 0.1807 |
| **Dual purpose IIa type** | 5 | **12** | Ar | 4.46 | 1 | 0.2448 |
| **Dual purpose IIa type** | 5 | **16** | PB | 4.16 | 1 | 0.3958 |
| **Dual purpose IIa type** | 5 | **18** | F | 4.07 | 1 | 0.3788 |
| **Dual purpose IIa type** | 5 | **23** | SL | 3.72 | 3 | 0.3701 |
| **Dual purpose IIa type** | 5 | **29** | ABS | 3.34 | 1 | 0.3494 |
| **Dual purpose IIa type** | 5 | **31** | Pm | 3.27 | 1 | 0.5285 |
| **Dual purpose IIa type** | 5 | **34** | OMF | 3.03 | 1 | 0.548 |
| **Dual purpose IIa type** | 5 | **36** | YC | 2.92 | 1 | 0.5011 |
| **Dual purpose IIa type** | 5 | **35** | UM | 2.92 | 1 | 0.403 |
| **Dual purpose IIb type** | 4 | **8** | LMF | 5.06 | 6 | 0.3805 |
| **Dual purpose IIb type** | 4 | **19** | PRB | 4.06 | 6 | 0.03519 |
| **Dual purpose IIb type** | 4 | **21** | Ts | 3.75 | 5 | 0.09229 |
| **Dual purpose IIb type** | 4 | **24** | PC | 3.72 | 5 | 0.1236 |
| **Dual purpose IIb type** | 4 | **30** | AoB | 3.31 | 5 | 0.3725 |
| **Dual purpose IIb type** | 4 | **37** | MG | 2.73 | 5 | 0.4385 |
| **Meat type** | 1 | **1** | CB | 10.06 | 6 | 0.5559 |
| **Meat type** | 1 | **2** | RWD | 8.57 | 6 | 0.5553 |
| **Meat type** | 1 | **5** | Pu | 5.29 | 6 | 0.4322 |
| **Meat type** | 1 | **6** | NH | 5.13 | 6 | 0.3454 |
| **Meat type** | 1 | **10** | LGG | 4.62 | 5 | 0.001174 |
| **Meat type** | 1 | **20** | ZS | 3.81 | 5 | 0.4433 |
| **Meat type** | 1 | **25** | SW | 3.70 | 5 | 0.4824 |
| **Meat type** | 1 | **32** | BB | 3.24 | 5 | 0.5467 |
| **Meat type** | 1 | **38** | UG | 2.48 | 5 | 0.5509 |
| **Meat type** | 1 | **39** | WC × (BL × SL) | 1.66 | 5 | 0.5509 |
| Mean *S* | | | | | | 0.3760 |
| SD (*S*) | | | | | | 0.1816 |

**n*, serial number based on descending sorting by mean *EY*/*W* values.

**The Silhouette measures how similar the object is to his cluster (-1: non-similar, 1: very similar).


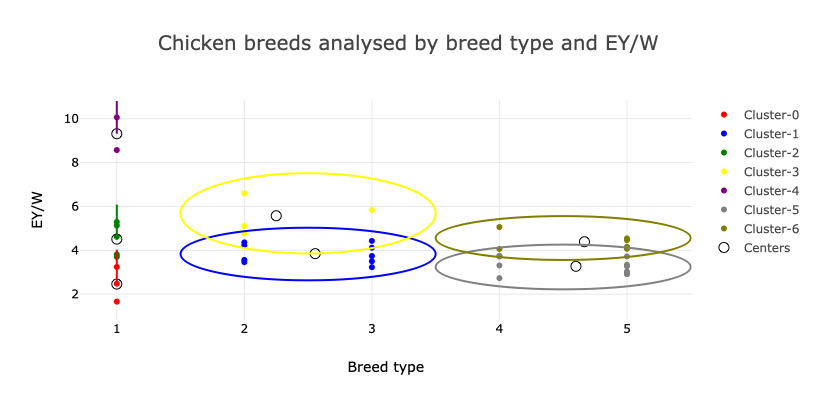


The data was divided into **7** clusters.
We chose the smallest k which explains at least 90% of the variance. (90.818%)
Clusters: **4,4,2,2,2,2,2,0,0,0,3,3,3,1,1,1,1,3,1,1,1,1,1,6,6,5,5,5,5,6,6,6,6,5,5,5,5,5,5**. (See the cluster column above)
Explained variance ratio: **0.9082**.
SSE - The sum of squared of the distances from all the points to the centers.
SSE (Within): **16.7502**.
SSG (Between groups): 165.674.
SST (Total): 182.4242.
SSE by group: 1.2488, 3.7912, 2.151, 2.7393, 1.1101, 3.6361, 2.0738.
Centers: [1,2.46], [2.5556,3.8478], [1,4.51], [2.25,5.575], [1,9.315], [4.6,3.271], [4.6667,4.3917].
Maximum iterations: 12.


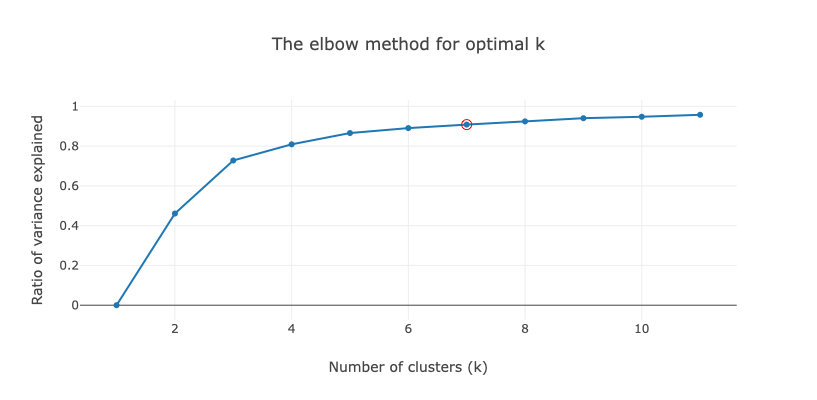


**9. Clustering of core breeds (as shown in Table below)**

| **Breed** | ***n**** | **Breed type code** | **Breed type by model** | | | | ***EY*/*W*** | **Silhouette**** |
| --- | --- | --- | --- | --- | --- | --- | --- | --- |
|  |  |  | **TCM** | **PCM** | | **GCM** |  |  |
| RWG | 4 | 1 | Egg | Egg | Egg Ib | | 5.83 | 0.4554 |
| LLB | 9 | 1 | Egg | Egg | Egg Ia | | 4.77 | 0.09507 |
| PB | 16 | 2 | Egg-meat | Egg-meat | Dual purpose IIa | | 4.16 | 1 |
| PRB | 19 | 3 | Meat-egg | Meat-egg | Dual purpose IIb | | 4.06 | 0.3283 |
| Ts | 21 | 3 | Meat-egg | Meat-egg | Dual purpose IIb | | 3.75 | 0.6145 |
| PC | 24 | 3 | Meat-egg | Meat-egg | Dual purpose IIb | | 3.72 | 0.6323 |
| ABS | 29 | 3 | Meat-egg | Meat-egg | Dual purpose IIa | | 3.34 | 0.7384 |
| AoB | 30 | 3 | Meat-egg | Meat-egg | Dual purpose IIb | | 3.31 | 0.7384 |
| Pm | 31 | 3 | Meat-egg | Meat-egg | Dual purpose IIa | | 3.27 | 0.7286 |
| YC | 36 | 3 | Meat-egg | Meat-egg | Dual purpose IIa | | 2.92 | 0.5888 |
| WC × (BL × SL) | 39 | 4 | Meat | Meat | Meat | | 1.66 | 1 |
| Mean *S* | | | | | | | 3.7082 | 0.6291 |
| SD (*S*) | | | | | | | 1.0612 | 0.2677 |

**n*, serial number based on descending sorting by mean *EY*/*W* values.

**The Silhouette measures how similar the object is to his cluster (-1: non-similar, 1: very similar).


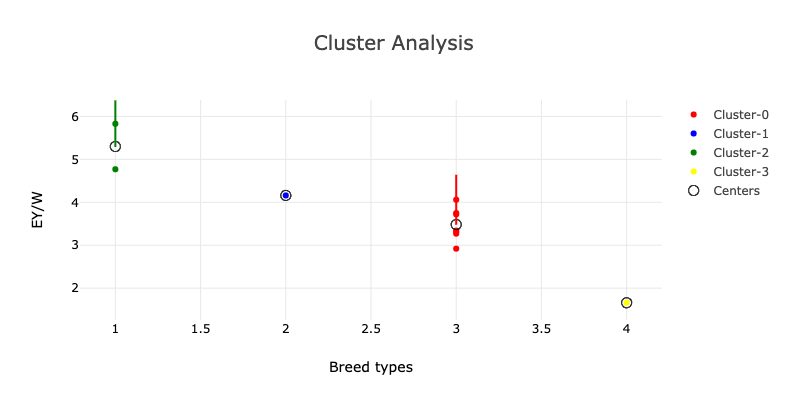


The data was divided into **4** clusters.
We chose the smallest k which explains at least 90% of the variance. (92.7557%)
Clusters: **2,2,1,0,0,0,0,0,0,0,3**. (See the cluster column above)
Explained variance ratio: **0.9276**.
SSE - The sum of squared of the distances from all the points to the centers.
SSE (Within): **1.4349**.
SSG (Between groups): 18.3723.
SST (Total): 19.8072.
SSE by group: 0.8731, 0, 0.5618, 0.
Mean ± SD (SSE by group): 0.3587 ± 0.4333

Centers: [3,3.4814], [2,4.16], [1,5.3], [4,1.66].
Maximum iterations: 5.


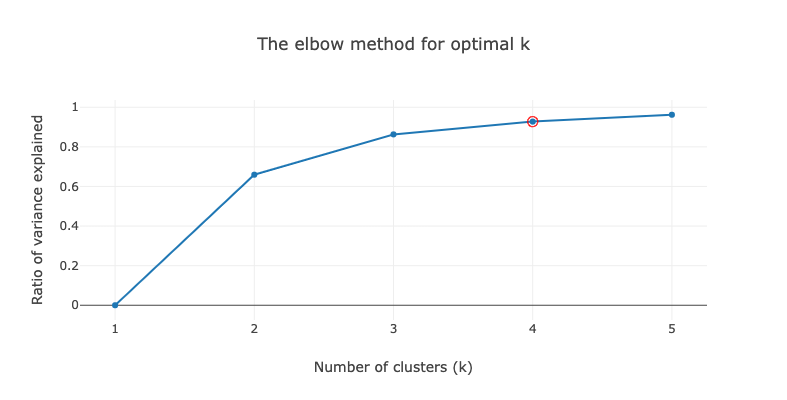

Supplement: Supplementary file 3 — Dataset S1. [file 41598_2023_28651_MOESM3_ESM.docx]
