# Supplementary material for: Disentangling clustering configuration intricacies for divergently selected chicken breeds
Source: Sci Rep. 2023 Feb 27;13:3319. doi: 10.1038/s41598-023-28651-8 (PMC9971033; doi:10.1038/s41598-023-28651-8)
Supplement: Supplementary file 5 — Dataset S3. [file 41598_2023_28651_MOESM5_ESM.docx]

**Supplementary data S3: Producing a curve for 39 chicken breeds**

When developing a curve for *EY*/*W* in hens of 39 breeds, the graph for this indicator was compared with that for body weight of males (Fig. S1-1). One can suggest that these two indicators are to some extent inversely correlated with each other.


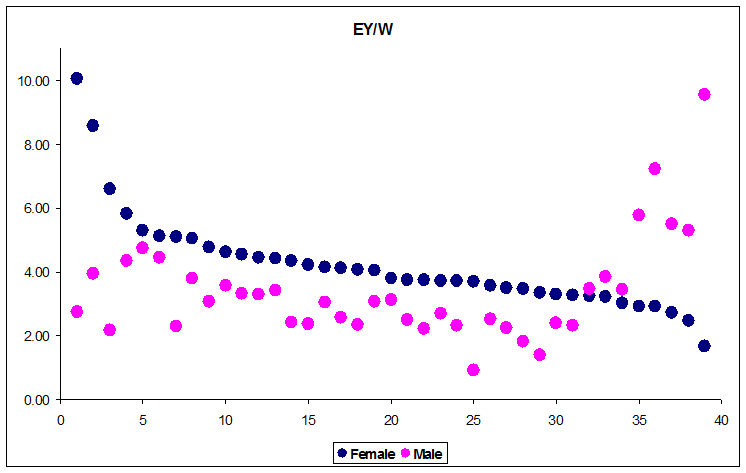


**Fig. S1-1.** Graph of the change in *EY*/*W* means (*y*-values) in females of 39 chicken breeds (*x*-values) relative to male body weight means.

Further, the egg productivity was recalculated by mass of the contents, assuming that the more a breed produces the internal edible part of the eggs (i.e., albumen and yolk), the more advantageous it can be considered in terms of egginess. According to Romanoff and Romanoff (1949), eggs of different weights have almost the same percentage of shell, i.e., around 10% (Fig. 1-2).


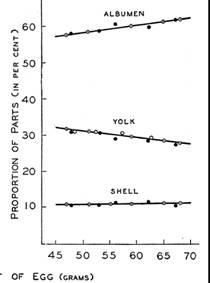


**Fig. S1-2.** Percentage of egg parts in chickens. The graph is adopted from: Romanoff AL, Romanoff AJ. The avian egg. New York: John Wiley & Sons Inc.; 1949.

Therefore, we multiplied the egg productivity by a factor of 0.9 and, as expected, got a graph in Fig. S1-3, similar to the female curve in Fig. S1-1.


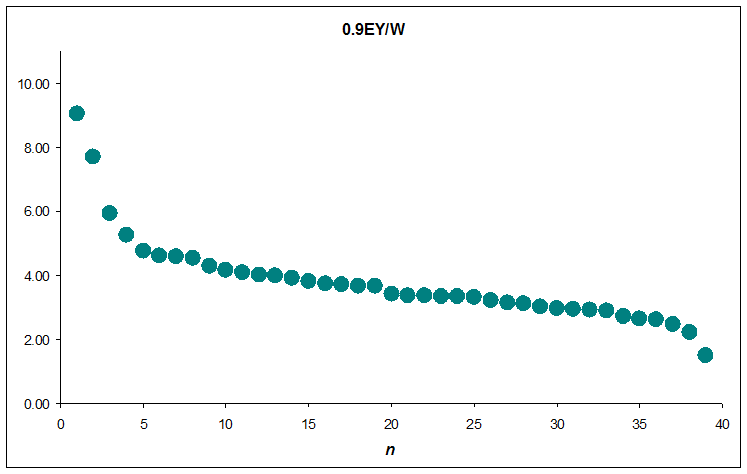


**Fig. S1-3.** Graph of the change in *EY*/*W* means multiplied by a factor of 0.9 (*y*-values) in females of 39 chicken breeds (*x*-values).

Therefore, the shell weight does not affect the resulting dependence and does not affect the subsequent clustering of chicken breeds relative to the *EY*/*W* index.
