# Supplementary material for: Disentangling clustering configuration intricacies for divergently selected chicken breeds
Source: Sci Rep. 2023 Feb 27;13:3319. doi: 10.1038/s41598-023-28651-8 (PMC9971033; doi:10.1038/s41598-023-28651-8)
Supplement: Supplementary file 6 — Dataset S4. [file 41598_2023_28651_MOESM6_ESM.docx]

**Supplementary data S4: Fitting a curve and defining inflection points for 11 core breeds of chickens**

A refined analysis for 11 core breeds was performed using the Statistica advanced analytics software package:

TIBCO Statistica, v. 12, TIBCO Software Inc, Palo Alto, CA, USA; 2013. Available from: https://www.tibco.com/products/tibco-statistica

Coordinates of 11 points:

*y*-values: 5.83 4.77 4.16 4.06 3.75 3.72 3.34 3.31 3.27 2.92 1.66

*x*-values: 1 2 3 4 5 6 7 8 9 10 11

or

(1, 5.83), (2, 4.77), (3, 4.16), (4, 4.06), (5, 3.75), (6, 3.72), (7, 3.34), (8, 3.31), (9, 3.27), (10, 2.92), (11, 1.66)

After fitting a curve, the respective trendline graph was obtained as shown in Fig. S2-1.

**Fig. S2-1.** Graph of the change in *EY*/*W* means (*y*-values) in females of 11 core breeds of chickens (*x*-values). The dataset was approximated with the respective trendline.

Polynomial-based trendline equation:

$\frac{EY}{W}=8.57394-3.91369n+1.4339n^{2}-0.26811n^{3}+0.0209n^{4}-0.00083n^{5}$, (1)

*R*² = 0.9962

Second derivative:

$\left(\frac{EY}{W}\right)^{''}=n^{3}-17.4778n^{2}+97.2674n-173.4013$ (2)

Equate the second derivative to 0.

$n^{3}-17.4778n^{2}+97.2674n-173.4013=0$ (3)

As a result of solving the cubic equation (3), the following roots were found:

*n*_1_ = 3.996

*n*_2_ = 5.311

*n*_3_ = 8.171

Inflection points: at *n*_1_ = 3.996, *n*_2_ = 5.311, and *n*_3_ = 8.171

Based on the above three inflection points, we obtained the following four clusters (with specific numbers of breeds in curly brackets given in accordance with their descending sequential sorting by *EY*/*W* values in the 39-breed model):

Cluster 1 (ETB): {4, 9, **16**}

Cluster 2 (EMB): {19, 21}

Cluster 3 (MEB): {23, 29, 30}

Cluster 4 (MTB): {31, 36, 39}

When comparing the inflection points models for 11 and 39 breeds, there was an almost perfect match. Each of the core breeds fell into the same proper interval as in the case of 39 breeds (Fig. 3). The only exception was the Pantsirevka Black breed. It had the 16th serial number in the large (i.e., 39-breed) model, meaning that it conditionally fell into the EMB category. In the reduced (i.e., 11-breed) model, it was among ETB. Yet, we believe that this was like a “borderline” case and, therefore, not a critical discrepancy. As one might notice and explain, the number of points used for fitting a curve could affect both the formula of the curve and the breakdown by inflection points.
